# Bridging the Gap in Cancer Research: Sulfur Metabolism of Leukemic Cells with a Focus on L-Cysteine Metabolism and Hydrogen Sulfide-Producing Enzymes

**DOI:** 10.3390/biom14070746

**Published:** 2024-06-24

**Authors:** Konrad Kaleta, Klaudia Janik, Leszek Rydz, Maria Wróbel, Halina Jurkowska

**Affiliations:** 1 Students’ Scientific Group of Medical Biochemistry, Faculty of Medicine, Jagiellonian University Medical College, 7 Kopernika St., 31-034 Krakow, Poland; konrad.kaleta@student.uj.edu.pl; 2 Chair of Medical Biochemistry, Faculty of Medicine, Jagiellonian University Medical College, 7 Kopernika St., 31-034 Krakow, Poland; klaudia.janik9768@gmail.com (K.J.); leszek.rydz@uj.edu.pl (L.R.); mtk.wrobel@uj.edu.pl (M.W.)

**Keywords:** leukemia, leukocytes, L-cysteine, sulfurtransferases, cystathionine beta-synthase, antioxidants

## Abstract

Leukemias are cancers of the blood-forming system, representing a significant challenge in medical science. The development of leukemia cells involves substantial disturbances within the cellular machinery, offering hope in the search for effective selective treatments that could improve the 5-year survival rate. Consequently, the pathophysiological processes within leukemia cells are the focus of critical research. Enzymes such as cystathionine beta-synthase and sulfurtransferases like thiosulfate sulfurtransferase, 3-mercaptopyruvate sulfurtransferase, and cystathionine gamma-lyase play a vital role in cellular sulfur metabolism. These enzymes are essential to maintaining cellular homeostasis, providing robust antioxidant defenses, and supporting cell division. Numerous studies have demonstrated that cancerous processes can alter the expression and activity of these enzymes, uncovering potential vulnerabilities or molecular targets for cancer therapy. Recent laboratory research has indicated that certain leukemia cell lines may exhibit significant changes in the expression patterns of these enzymes. Analysis of the scientific literature and online datasets has confirmed variations in sulfur enzyme function in specific leukemic cell lines compared to normal leukocytes. This comprehensive review collects and analyzes available information on sulfur enzymes in normal and leukemic cell lines, providing valuable insights and identifying new research pathways in this field.

## 1. Introduction

Sulfurtransferases (EC 2.8.1.) are enzymes that transfer sulfur-containing groups from a sulfur donor to a nucleophilic sulfur acceptor. These enzymes have been the subject of interest for decades within our department. One of the most notable members of this group, 3-mercaptopyruvate sulfurtransferase (MPST, EC 2.8.1.2), is closely linked to human sulfur metabolism. MPST, along with cysteine aminotransferase (CAT, EC 2.6.1.3), and cystathionine γ-lyase (CTH, EC 4.4.1.1) play a key role in hydrogen sulfide (H_2_S) metabolism. H_2_S is a signaling gaseous transmitter that was discovered in 1996 when its formation in brain tissue by cystathionine β-synthase (CBS, EC 4.2.1.22) was first demonstrated [1,2].

Three of these enzymes—MPST, CBS, and CTH—are commonly considered principal in H_2_S production. However, their action also intersects with other fundamental intracellular pathways such as cysteine and methionine metabolism, energy metabolism, and the sulfur relay system [1,2].

Three of these enzymes—MPST, CBS, and CTH—are thus considered essential for proper cellular function. Additionally, thiosulfate sulfurtransferase (TST, also known as rhodanese, EC 2.8.1.1), which is evolutionarily, chromosomally, and phenotypically similar to MPST, will be the focus of this study. For the purposes of this study, all four enzymes are tentatively referred to as H_2_S-metabolizing enzymes.

**Figure 1:**
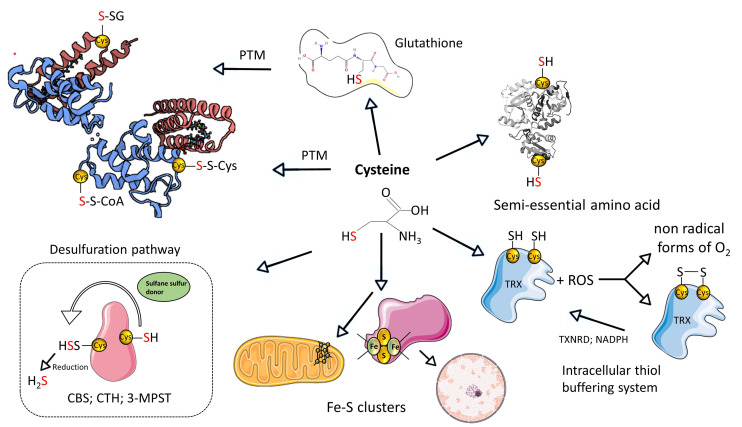
Summary of the L-cysteine metabolism. Created based on references [10,11]. Abbreviations: [SH]—thiol group; [PTM]—post-translational modifications; [-S-SG]-S-glutathionylation; [-S-S-Cys]-S-cysteinylation; [-S-S-CoA]-S-CoAlation; H_2_S—hydrogen sulfide; [ROS]—reactive oxygen species; [CTH]—cystathionine-γ-lyase; [3-MPST]—3-mercaptopyruvate sulfurtransferase; [CBS]—cystathionine beta synthases; [TRX]—thioredoxin; [TXNRD]—thioredoxin reductase. Illustrations within figure are adapted from Servier Medical Art (https://smart.servier.com), accessed in February 2024, courtesy of Servier, under a Creative Commons Attribution 4.0 Unported License available at https://creativecommons.org/licenses/by/4.0/ accessed in February 2024.

Cysteine metabolism constitutes crucial biochemical pathways essential for the sustenance and functioning of a full range of live forms. These pathways are intricately linked to the metabolism of sulfur-containing amino acids. L-cysteine, a vital amino acid integral for all living organisms, is distinguished by its thiol (-SH) group, which is essential to maintaining cellular redox equilibrium. It is a foundational element in the synthesis of major cellular antioxidants such as glutathione (in its reduced form, GSH, and oxidized form, GSSG), glutaredoxins, thioredoxins, and peroxiredoxins, all vital for protecting cells against oxidative stress [3,4,5,6]. The synthesis of L-cysteine within cells primarily involves the transformation of methionine and homocysteine through the transsulfuration pathway [7]. This pathway relies on the activity of the cytosolic pyridoxal-5′-phosphate (PLP)-dependent enzymes: cystathionine β-synthase CBS and cystathionine-γ-lyase CTH (Figure 1).

**Figure 2:**
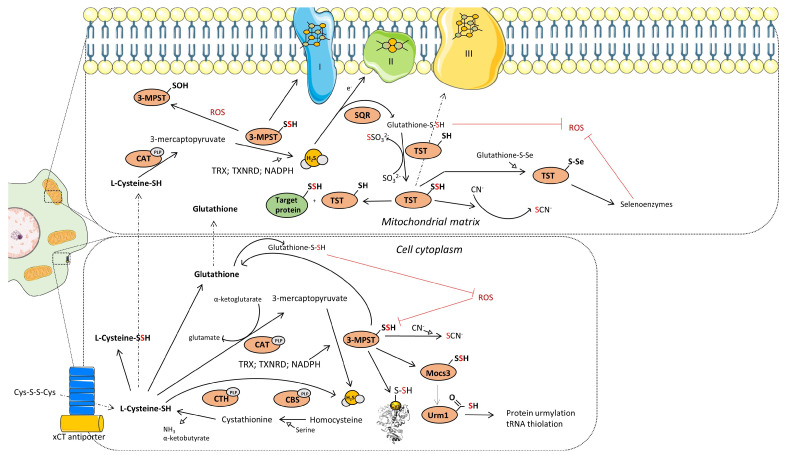
Schematics representation of the metabolic pathways that contribute to H_2_S generation in cytoplasm and mitochondria. Mechanism of transsulfuration (TSS) pathway, SQR:TST and CAT:MPST enzymatic axis. Created based on reference [12]. Abbreviations: [xCT antiporter]—cysteine/glutamate transporter; [CTH]—cystathionine-γ-lyase; [CBS]—cystathionine beta synthases; [PLP]-pyridoxal-5′-phosphate; [3-MPST]—3-mercaptopyruvate sulfurtransferase; [CAT]—cysteine aminotransferase; [ROS]—reactive oxygen species; [Mocs3]-molybdenum cofactor synthesis 3; [Urm1]-ubiquitin related modifier 1; [TST]—thiosulfate sulfurtransferase; [SQR]—sulfide quinone reductase; [TRX]—thioredoxin; [TXNRD]—thioredoxin reductase. Illustrations within figure are adapted from Servier Medical Art (https://smart.servier.com), accessed in February 2024, courtesy of Servier, under a Creative Commons Attribution 4.0 Unported License available at https://creativecommons.org/licenses/by/4.0, accessed in February 2024.

As previously mentioned, these enzymes are not only crucial for L-cysteine production but also for the generation of H_2_S, a compound of significant biological and pharmacological importance [8]. MPST, in tandem with CAT and in the presence of α-ketoglutarate, can also utilize L-cysteine to produce H_2_S. Evolutionarily, genetically, and functionally related to MPST is the enzyme TST, which plays a vital role in H_2_S and cyanide detoxification. TST, in particular, serves as an essential link between the detoxification of cyanide and the metabolism of organosulfur compounds, underscoring the interconnected nature of detoxification, antioxidation, and sulfur metabolism [9] (Figure 2).

Previous laboratory investigations have identified dysfunctions in these four key enzymes as critical contributors to various cancers and malignancies.

Empirical studies have demonstrated a robust correlation between the proliferation of specific cancerous cell lines, notably human astrocytoma (U373), glioblastoma (U87MG), and neuroblastoma (SH-SY5Y), and the expression and activity of sulfurtransferases. The levels of sulfane sulfur within these cells have been closely associated with these pathological states [13,14,15,16]. Recently, the transsulfuration pathway, which involves the utilization of homocysteine and the production of hydrogen sulfide, has been identified as a promising target for cancer treatment [17]. Despite these advances, our understanding of the presence, functionality, and expression levels of these enzymes in leukemia cells is still limited.

Leukemia, a neoplastic disease primarily affecting the bone marrow—the critical site for hematopoietic stem cells—leads to the excessive production of aberrant cancer cells in the bloodstream [18]. While the precise etiology of leukemia remains elusive, various environmental and genetic factors have been identified as potential contributors to this neoplastic process [19].

Leukemia encompasses a range of blood malignancies that arise from mutations in developing leukocytes or hematopoietic stem cells. While it remains the most prevalent cancer type in children, adults are also significantly affected by this disease [19]. The disease is categorized by both the type of affected blood cells (lymphoblastic/lymphocytic or myeloid) and the rate of progression (acute or chronic). The latest 5th edition of the WHO Classification of Haematolymphoid Tumors provides detailed differentiation among various leukemia types [20,21]. However, for the purposes of this article, we will adopt the classification system used by the Surveillance, Epidemiology, and End Results Program, based on the earlier FAB classification. This system delineates four primary leukemia types: Acute Lymphoblastic Leukemia (ALL), Acute Myeloid Leukemia (AML), Chronic Lymphocytic Leukemia (CLL), and Chronic Myeloid Leukemia (CML) [18].

The landscape of leukemia treatment has witnessed significant advancements, as evidenced by a ten-year survival rate that varies between 40 and 86% depending on the leukemia subtype [22]. Bone marrow transplantation, targeted therapy, and the innovative chimeric antigen receptors T-cell (CAR-T) therapy have revolutionized the approach to this disease, with an anticipated increase in patient survival rates [23]. Despite these advancements, several therapeutic challenges persist, including issues like treatment resistance, adverse side effects, and the compatibility of bone marrow donors [24,25]. In many cases, complete remission remains elusive, and patients may progress to a state of minimal residual disease, where leukemia cells are undetectable by standard diagnostic tests. This scenario can result in the selection of treatment-resistant leukemia cell clones, potentially leading to disease relapse with severely limited treatment options [26]. All of these factors underscore the need for research into new therapeutic techniques that can either further extend patients’ lives or offer complete recovery. In light of these challenges, our laboratory’s recent investigations into specific enzymes in leukemic cell lines have yielded promising results [27]. This paper aims to offer an in-depth review of the current literature, focusing specifically on the role of enzymes in sulfur metabolism within leukemic cells. It seeks to identify potential bottlenecks in sulfur metabolism in leukemia cells, concentrating on four key enzymes: TST, MPST, CTH, and CBS. The goal is to enhance understanding of the subject and serve as a foundation for further empirical research into this area, exploring their potential as targets for future therapeutic interventions and identifying new areas for study.

## 2. Sulfurtansferases and Cystathionine β-Synthase in Normal Leukocytes

Current knowledge regarding the expression and activity of enzymes involved in L-cysteine metabolism within white blood cells is limited. Nonetheless, the scant available information provides insights into specific trends and offers valuable building blocks for interpreting this topic. To fill in the missing information, we utilized several online databases, such as BioGPS (http://biogps.org/about) accessed in December 2023 [28] and the Immunological Genome Project (https://www.immgen.org/, accessed in December 2023) [29]. The results from these databases are presented below.

### 2.1. 3-Mercaptopyruvate Sulfurtransferase and Thiosulfate Sulfurtransferase

One of the first studies that examined the activity of the MPST enzyme in human leukocytes was performed by Mårtensson and Sörbo [30] with the use of the established Valentine and Frankenfeld method [31]. In the blood specimens from healthy subjects, it was demonstrated that the enzyme activity was significantly lower in granulocytes (0.037 ± 0.013 mkat/L cells) and lymphocytes (0.11 ± 0.04 mkat/L cells) in comparison to erythrocytes (1.68 ± 0.17 mkat/L cells) and platelets (2.11 ± 0.43 mkat/L cells) [30]. It was demonstrated that even in healthy individuals, MPST activity is the lowest in the white blood cells when compared to other blood cells.

**Figure 3:**
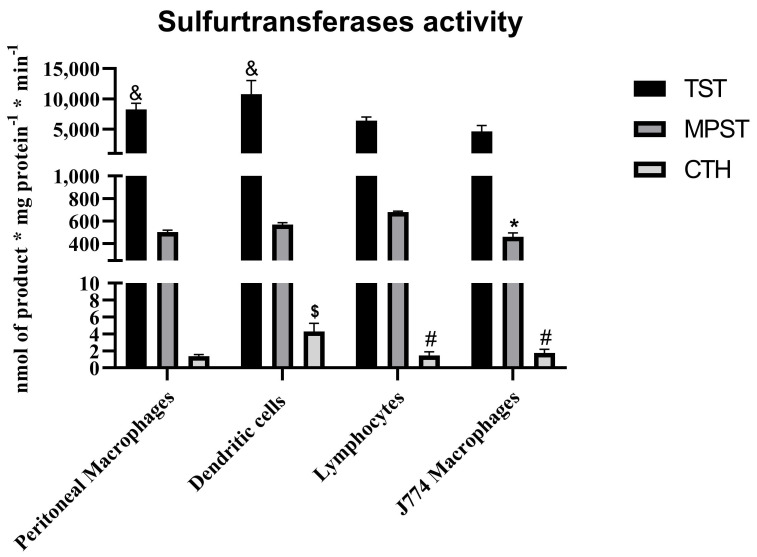
The sulfurtransferases activity within the immune cells from Balb/c mice and J774 murine macrophage cell line—based on results published by Wróbel et al. [32]. *p* < 0.05—MPST: lymphocytes vs. J774 line (*); CTH: macrophages vs. dendritic cells ($), dendritic cells vs. J774 line (#), dendritic cells vs. lymphocytes (#); TST: lymphocytes vs. macrophages (&), lymphocytes vs. dendritic cells (&).

There was a lack of validating studies about TST expression and/or activity within leukocytes. Our interpretation can be based on the work performed by Wróbel et al. [32] giving us a complex picture of the activity of sulfurtransferases in murine immune cells (shown in Figure 3). It was found that CTH activity is rather negligible within these cells, in contrary to MPST and TST.

Based on mouse expression patterns (Table 1), *Tst* expression in most leukocytes appears to be at a consistent, medium level. Activation, physiological transformations, and maturation seem to lead to only minor changes in *Tst* expression. Peritoneal macrophages (thio-elicited day 5) exhibit exceptionally high levels of *Tst* (319.041) and *Mpst* (208.446), indicating enhanced sulfur metabolism during immune activation. Interestingly, bone marrow neutrophils have the highest *Tst* expression (599.476) among all cell types, suggesting a significant role for *Tst* in granulocyte function. *Mpst* expression is elevated in dendritic cells, certain T-cell populations, and activated macrophages. Splenic NK cells, including subsets, show consistent *Tst* (around 90) and *Mpst* (around 100) levels, indicating stable sulfur/H_2_S metabolism across different NK cell populations.

Based on human expression patterns (Table 2) of TST and MPST, we observe that naive B-cells (B.NveIgD^+^27) exhibit moderate TST (10.7159) and high MPST (112.302) expression levels, indicating active sulfur metabolism. In contrast, memory B-cells (B.MemIgD^−^27^+^38) show significantly lower TST (2.78259) and MPST (40.7265) expression, suggesting reduced sulfur metabolism in these cells. Immature NK cells (ILC.Nkimm.56hi16) have the highest MPST expression (183.559) among all ILCs, indicating intense sulfur metabolism during early NK cell development. Naive CD4 T-cells (T.4Nve.CD3^+^4^+^RA^+^62L) show low TST (4.13621) and MPST (6.54517) levels, whereas effector memory CD4 T-cells (T.4EffMem.CD3^+^4^+^RA^−^62L) demonstrate higher TST (10.307) and MPST (39.5897) expression. Similarly, effector memory CD8 T-cells (T.8EffMem.CD3^+^8^+^RA^−^62L) exhibit significantly higher MPST (84.3976) levels, suggesting enhanced sulfur metabolism during effector functions. Interestingly, resting Tregs (T.Treg.rest) have low TST (2.51367) and MPST (11.5957) levels, while activated Tregs (T.Treg.act) show increased TST (4.71504) and a 10-fold increase in MPST (122.602), indicating metabolic adaptation upon activation. DC1 cells (DC.DC1.141) present high TST (61.2666) and MPST (249.952) levels, reflecting a robust metabolic profile. Among specialized DCs, DC5 cells (AXL^+^SIGLEC6) display unique metabolic profiles with the highest TST and significant MPST expression.

**Figure 4:**
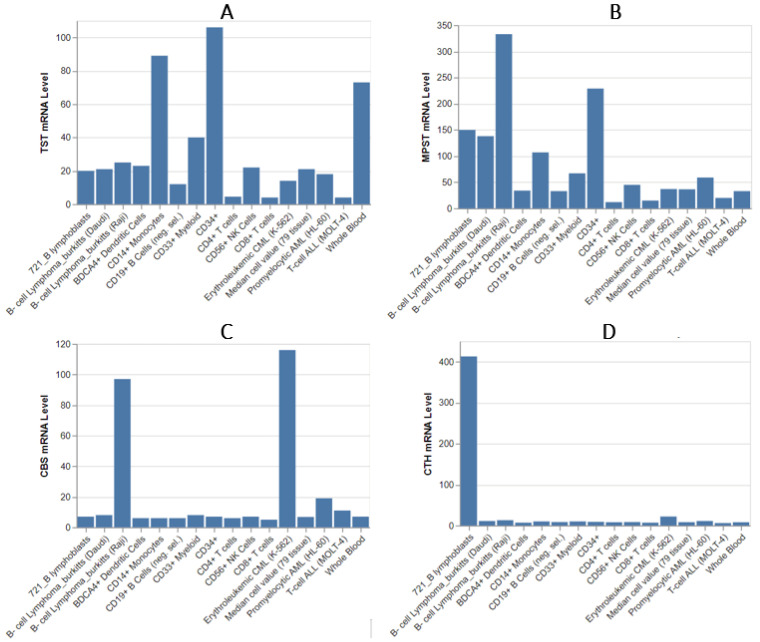
The mRNA levels of TST (**A**), MPST (**B**), CBS (**C**), and CTH (**D**) among leukocytes and some lymphoid cancer cell lines. This dataset was compiled using high-density oligonucleotide arrays and is sourced from the BioGPS Library (http://biogps.org/about/), accessed in December 2023 [28].

The analysis of TST expression (Figure 4) using data from BioGPS [28] revealed that the highest TST mRNA levels are found in CD34^+^ cells. High TST levels are also observed in CD14^+^ cells and whole blood. Other cell types, including erythroid cells and CD56^+^ NK cells, exhibit lower TST mRNA levels. TST expression is low in most B-cells, T-cells, and myeloid cells. Interestingly, despite the typical expression levels of TST in most cell populations, an enhanced expression of MPST is observed in B-lymphoblasts and B-cell Burkitt lymphomas. For instance, in the Raji cell line, MPST expression is seemingly 10-fold higher compared to normal B-cells. Burkitt lymphoma, a highly aggressive non-Hodgkin B-cell lymphoma often associated with Epstein–Barr virus infection, is characterized by rapid growth. Therefore, the observed high MPST expression pattern in this data could be correlated with increased cell division and proliferation, as also suggested by its expression in the CD34^+^ cell population.

In conclusion, MPST expression is generally higher across most leukocyte populations compared to TST, indicating its prominent role in sulfur metabolism in immune cells. Notably, MPST has the highest expression levels among all studied enzymes. An especially interesting observation is the very high expression levels of TST and MPST in phagocytic cells such as dendritic cells (DCs) and monocytes, as confirmed by data from BioGPS [28], the Immunological Genome Project [29], and the study by Wróbel and colleagues [32]. This effect is also observable in other datasets, such as the Human Protein Atlas [https://www.proteinatlas.org/] [34,35]. Not all phagocytic cells exhibit such high expression levels. For example, neutrophil results are ambiguous, showing either exceptionally high expression levels as seen in mouse data or levels comparable to other leukocytes. This variability may be related to the need for oxidative protection against self-damage caused by oxidative bursts, necessitating further research. TST expression is generally high not only in granulocytes and certain macrophages but also in hematopoietic stem cells (HSCs). Similarly, immature ILCs and naive B-cells exhibit higher MPST and TST levels than their mature and specialized counterparts, suggesting a significant role for these enzymes during development. Given that MPST and TST are involved in the construction of iron–sulfur centers, they may play a critical role during cell division. Conversely, naive T-cells and resting Tregs show lower TST and MPST expression, reflecting their relatively quiescent metabolic state. Notably, TST and MPST expression levels are relatively low in lymphocyte populations, including mature B-cells, CD4^+^ T-cells, and CD8^+^ T-cells.

### 2.2. Cystathionine β-Synthase and Cystathionine Gamma-Lyase

Early investigations by Goldstein et al. [36] detected no CBS enzyme activity in non-activated leukocytes, with only trace activity observed in lymphocytes. This activity significantly increased following phytohemagglutinin stimulation [36]. Additionally, Allsop and Watts [37] evaluated the activity of transsulfuration pathway enzymes, including S-adenosylmethionine synthetase, CBS, and CTH, in human polymorphonuclear leukocytes (PMC), lymphocytes, and monocytes. They reported an absence of activity for these enzymes within the cells examined. Subsequent research has primarily focused on CBS activity in stimulated peripheral blood mononuclear cells (PBMCs). Later studies identified CBS mRNA in resting lymphocytes, suggesting potential trace cellular activity [38]. A significant advancement was made by Katko et al. [39], who demonstrated CBS expression and activity in resting PMCs from eight healthy donors. They reported a mean CBS activity of 0.99 ± 0.32 mU/mg protein, ranging from 0.6 to 1.59 mU/mg protein. The study found that CBS protein levels and activity increased with incubation time and upon stimulation, influenced by intra- and extracellular changes in homocysteine and GSH concentrations, both in vitro and in vivo [39].

Garg et al. [40] made a noteworthy discovery when Western blot analyses showed that CBS and methionine synthase were undetectable in monocytes but became inducible during differentiation. It was established that the expression of CBS and methionine synthase is significantly upregulated during the differentiation of human monocytes, regulated at both transcriptional and post-transcriptional levels [40]. The inhibition of the transsulfuration pathway with propargylglycine reduced the clearance of Mycobacterium smegmatis by macrophages and obstructed phagolysosomal fusion. Conversely, N-acetylcysteine facilitated phagolysosomal fusion and tripled mycobacterial clearance compared to untreated cells. This suggests that modulation of the transsulfuration pathway during monocyte differentiation, activation, and infection can enhance host defenses against pathogens and may represent an unexplored therapeutic avenue for antimicrobial treatment [40].

Silencing the CTH gene in monocytes using siRNA has been shown to protect mice from caerulein-induced acute pancreatitis and concurrent lung injury [41]. This protection is evidenced by both biochemical markers (such as plasma amylase levels and myeloperoxidase activity in pancreatic and lung tissues) and histological analysis (hematoxylin and eosin staining of tissue sections). The mechanism appears to involve a reduction in cytokine levels within the pancreas and lungs. These findings highlight the significant role that the CTH enzyme may play during the inflammatory process. Additionally, CTH gene silencing seemed to prevent the reduction in splenic monocyte numbers observed in the acute pancreatitis model, suggesting that the spleen may serve as a reservoir for monocytes during inflammation. This implies that CTH-derived H_2_S might be crucial for communication between immune cells, and inhibiting CTH in active leukocytes could disrupt this signaling pathway [41].

Li et al. [42] found that CTH is expressed in rat neutrophils and that its expression is upregulated by lipopolysaccharide (LPS). Notably, co-incubation with dexamethasone mitigated the LPS-induced increases in TNF-α, IL-1β, and L-selectin expression. Dexamethasone also suppressed CTH (and iNOS) expression in LPS-challenged neutrophils and in human fetal liver cells. The use of QNZ, an inhibitor of NF-κB transcriptional activation, abrogated the rise in CTH expression in neutrophils, enhancing the evidence that NF-κB substantially regulates CTH expression. This study further supports the hypothesis that dexamethasone reduces tissue H_2_S formation by inhibiting NF-κB activation through a steroid receptor-dependent pathway. This was possibly the inaugural report of endogenous H_2_S release from neutrophils, which are known to be influenced by exogenous H_2_S. Notably, this study provided the first evidence of endogenous H_2_S release from neutrophils, suggesting that H_2_S may function as an autocrine mediator in neutrophils, which could be affected by dexamethasone’s modulation of H_2_S and nitric oxide production [42].

The significant role of CTH in immune system regulation has also been corroborated by other high-impact studies [43,44,45]. However, these studies have not yet elucidated the specific expression and activity of CTH, pointing to an area for further research.

Regarding CBS and CTH expression in leukocytes (Table 1 and Table 2), it is generally very low or even trace. CBS expression is consistently low across all leukocyte populations, suggesting a lesser role in sulfur metabolism compared to TST and MPST. The highest expression of both CBS and CTH is present among naive B-cells. Elevated CBS levels in certain B-cell populations (e.g., spleen follicular B-cells) and memory T-cells suggest its role in supporting cellular proliferation and function.

Significant expression CBS (Figure 4) mRNA level is also observed in one subtype of Burkitt lymphomas. Low to negligible CBS mRNA levels are found in most other leukocyte types, including CD34^+^ cells, CD8^+^ T-cells, and myeloid cells, with expression levels similar to those in other tissues. Whole blood and CD19^+^ B-cells show very low CBS expression.

This is a significant observation that warrants further investigation because it may indicate that overexpression of CBS and/or CTH is closely associated with the pathogenesis of cancer. This is evidenced by the markedly increased expression of CTH in 721 B-lymphoblasts and CBS in lymphoid and malignant cell lines (K562, Raji).

The research results find consistency with our previous work by Jurkowska et al. [27]. The expression of TST in MOLT-4 was practically absent, which aligns with the data presented in Figure 4. However, given the similarly minimal expression in normal CD4^+^ T lymphocytes, as confirmed by different data, this value is expected and indicates that the pathology does not affect expression. Similar observations can be made for MPST and the MOLT-4 cell line. The significantly higher expression of TST and MPST in the K562 cell line compared to MOLT-4 is also confirmed. In the experiment, the K562 cell line exhibited the highest expression of CBS among all tested lines, which is also corroborated by database information. The K562 line demonstrated the highest expression of all four studied enzymes, which is again confirmed by the data. This is quite interesting, as it may suggest a sulfur/cysteine dependency among certain CML subtypes.

### 2.3. Insights and Constraints Regarding the Current Understanding of Sulfurtransferases in Immune Cells

Firstly, there is a notable lack of information regarding the expression profiles of other types of immune cells, such as eosinophils, basophils, and mast cells. These cells, or their derivative forms, can also be involved in leukemia. Furthermore, individual types of immune cells can exhibit different genetic profiles within their subpopulations, a topic that warrants further exploration.

The literature also indicated that significant changes in the expression of these enzymes can also arise in the case of cell activation to fulfill its designated role in the body—an interesting aspect here seems to be the regulation of CTH expression by the NF-κB factor and the role H_2_S may play in the communication of immune cells as was proposed in the work of Li et al. [42]. Moreover, not only the presence of infectious factors but also the occurrence of chronic diseases in patients may affect the activation of immune system cells and therefore the expression of these enzymes. Further work should particularly focus on studying the expression and activity of TST and MPST among immune system cells, due to significant gaps in the literature about their expression.

**Figure 5:**
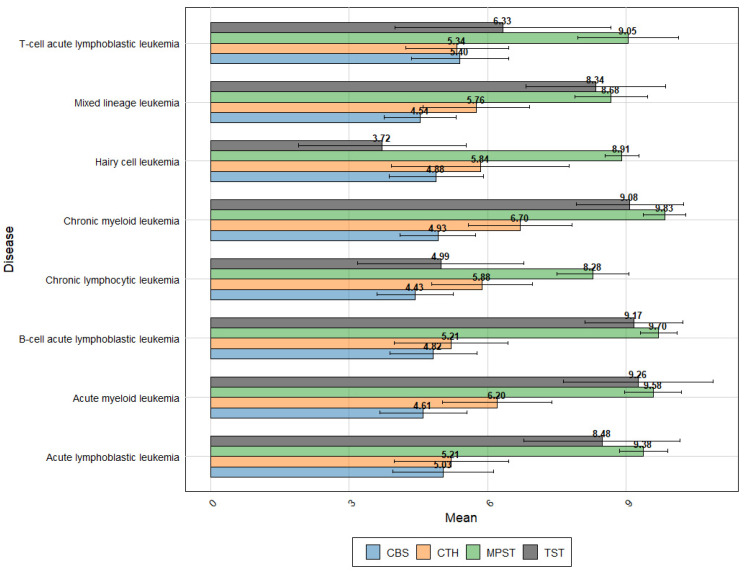
The expression profiles of key enzymes—TST, MPST, CTH, and CBS—across various leukemic subtypes. This analysis is based on data sourced from the Gene Expression Database of Normal and Tumor tissues (GENT2 platform-http://gent2.appex.kr/gent2), accessed in June 2024 [46].

The leukemic expression profiles obtained from the GENT2 platform [46], as shown in Figure 5, demonstrate significant variability in expression patterns among pathological leukocytes. These data not only highlight the differences but also further confirm some of current observations. The expression pattern of H_2_S-metabolizing enzymes in T-cell ALL shows consistent results when compared to the expression data of normal T-cells. Notably, the particularly high expression of CTH observed in B-lymphoblasts (Figure 4) is not corroborated by the current data. The data suggest a significant increase in TST expression in B-cell Acute Lymphoblastic Leukemia compared to T-cell Acute Lymphoblastic Leukemia (*p* < 0.001, Log2FC = 2.789). Interestingly, Chronic Myeloid Leukemia (CML) exhibits the highest expression levels of MPST and CTH, along with very high TST expression.

Overall, Acute Lymphoblastic Leukemia shows significantly higher TST expression compared to chronic lymphocytic leukemia (CLL) (*p* < 0.001, Log2FC = −3.421). Similarly, MPST (*p* < 0.001, Log2FC = −1.098) and CBS (*p* < 0.001, Log2FC = −0.582) also exhibit higher expression levels in Acute Lymphoblastic Leukemia, whereas CTH expression is lower (*p* < 0.001, Log2FC = 0.644) (Figure 5).

In fast-proliferating lymphocytic leukemias, there appears to be an increased demand for H_2_S-metabolizing enzymes crucial for proper sulfur metabolism, which aligns with the high expression observed in CD34^+^ stem cells. Intriguingly, this effect is not as pronounced in myeloid leukemias. The difference in TST expression is particularly notable, further confirmed by data from the CLL subtype—Hairy cell leukemia—which has the lowest TST expression among all the diseases studied.

It is also worth noting the higher CBS expression in acute lymphocytic leukemia compared to chronic leukemias. While it is challenging to compare the expression values of different genes directly without normalization, these findings could support the hypothesis of the predominant role of MPST expression in both physiological and pathological leukocytes.

In summary, the study highlights the distinct expression patterns of H_2_S-metabolizing enzymes across various types of leukemia, suggesting a potential link between enzyme expression and disease proliferation rates. Further research is necessary to fully understand the implications of these findings.

## 3. Sulfurtansferases and Cystathionine β-Synthase in Various Types of Leukemia

### 3.1. Thiosulfate Sulfurtransferase in Various Types of Leukemia

Thiosulfate sulfurtransferase (rhodanese) is a mitochondrial enzyme that is involved in several physiological functions in humans, including cyanide detoxification, formation of iron–sulfur centers, participation in energy metabolism, thiamin biosynthesis, selenium and sulfane compounds metabolism and functioning as thioredoxin oxidase [9] (Figure 2). It is worth noting the presence of the rhodanese domain, also known as the rhodanese homology domain, in a wide variety of enzymes, especially those involved in sulfur metabolism and signaling. The rhodanese domain can be found in approximately 500 proteins across different organisms, including at least 47 in humans. In the context of cancer cells, the rhodanese domain’s presence in phosphatases such as the Cdc25 family is particularly noteworthy, as these enzymes help regulate the cell cycle. While the rhodanese domain typically aids in catalyzing sulfur transfer reactions in other enzymes, its specific function can vary depending on the protein it is a part of and the overall protein structure [47]. Despite the importance of this enzyme and proven variable expression as well as activity in multiple types of cancers, there is little information in the accessible literature regarding rhodanese in leukemic cells.

Gal and colleagues conducted a study in 1952 [48] examining the expression and activity of rhodanese in various cancerous cell lines from different organs transplanted into mice. They analyzed myeloid leukemia cells transplanted into C57 mice and found no significant difference in rhodanese content between the malignant cells and mouse organs [48]. In a different study, Koeffler et al. [49] treated two distinct AML cell lines, KG-1 and HL-60, with amygdalin (a source of cyanide). They discovered no significant disparity in viability between leukemia lines and bone marrow cells, implying no difference in the cyanide detoxification process. It is noteworthy that actively phagocytic cells can liberate endogenous HCN to counteract pathogens, underscoring the necessity for an effective anti-cyanide defense system within these cells [50,51]. Given that rhodanese forms a significant part of the body’s defense against cyanide, this evidence strongly suggests no difference in rhodanese expression and activity in these AML cell lines and normal bone marrow cells [49]. Furthermore, our previous research aligns with these findings, as we discovered unchanged rhodanese expression in all examined AML (MOLM-14, MV4 cell lines) and CML (K562 cell line) cells. However, rhodanese was not expressed in T-cell Acute Lymphoblastic Leukemia (T-cell ALL) cell lines (DND-41 and MOLT-4) [27]. The results concerning the MOLT-4 cell line are consistent with the data presented on Figure 4.

Interestingly, in the field of machine learning, the TST gene was identified as one of the valuable genes detectable through microarray data from several patients for distinguishing acute leukemias. Dubitzky et al. [52] decision tree analysis pinpointed it as one of the 16 influential genes that could be used to differentiate between ALL and AML. Moreover, Sewak’s neural network model listed the TST gene among the top 250 genes capable of distinguishing three classes of leukemias (T-cell ALL, B-cell ALL, and AML) [53]. These bioinformatics observations add another layer of confirmation to our results, suggesting that further research on rhodanese expression in leukemic cells is highly recommended.

### 3.2. 3-Mercaptopyruvate Sulfurtransferase: Cysteine Aminotransferase Axis in Various Types of Leukemia

Human MPST, consisting of 297 amino acid residues, forms a ~33 kDa monomer featuring two rhodanese-like domains: the N-terminal (residues 1–138) and C-terminal (residues 165–285), connected by a 26-amino acid linker. These domains, which may have evolved from gene duplication, are crucial for MPST’s structure and function, notably since the C-terminal domain is the only functional one [54]. The enzyme is present both in the cytosol and mitochondria [55,56] is involved in L-cysteine transformation in cooperation with PLP-dependent cysteine aminotransferase encoded by glutamic-oxaloacetic transaminase (*GOT*) gene as a part of MPST:CAT enzymatic axis [56,57,58]. Similar to MPST, two CAT isoforms are distinguished depending on their cellular localization [57]. In progress of the axis, CAT transform L-cysteine to provide 3-mercaptopyruvate (3MP); however, a possible alternative pathway to provide 3MP exists [58].

MPST catalyzes the transformation of 3-mercaptopyruvate into pyruvate, concurrently shifting a sulfur atom to an acceptor like cyanide to generate thiocyanate or glutathione and cysteine to form proper persulfides [59] or directly generates H_2_S in the presence of reducing agent such as thioredoxin [60] (Figure 2). Beyond cyanide detoxification, MPST plays a role in a series of transformations that lead to protein urmylation and tRNA thiolation [58]. Interestingly, its activity can influence a cell’s antioxidative defense [61,62] and potentially shape the tumor microenvironment [63]. Moreover, it can function as a protein persulfidase [64]. Hydrogen sulfide, notably, can modify the thiol group of L-cysteine through the addition of sulfane sulfur (S-sulfuration), either directly or after oxidation as a polysulfide. These transformations are often linked to stress-sensing events, leading to changes in protein conformation or oligomerization [65,66,67,68]. The activity of MPST is regulated by several factors, including the concentration of calcium ions (Ca^2+^), the redox environment, oxidative stress, and the prodrugs of 3-mercaptopyruvate [69,70,71,72,73].

The protein level of MPST, found to be significantly upregulated in a variety of cancers, serves multiple complex roles, profoundly influencing cancer cell growth, proliferation, and resistance to oxidative stress [74,75]. Intriguingly, the activity of CAT: MPST enzymatic axis is dependent on the GSH level and the presence of L-cysteine [76]. Moreover, the MPST activity may also be altered by the presence of thioredoxin reductase and the glutaredoxin-reducing system [77].

Despite the absence of significant differences in mRNA and protein levels of MPST in Chronic Myeloid Leukemia K562 cells, a statistically significant difference was found in H_2_S levels compared to human CD34^+^ umbilical cord hematopoietic stem cells [78]. Various leukemia cell lines (REH, MV4-11, MOLM-14, and K562) have shown higher mRNA and protein levels of MPST, compared to cells from T-ALL leukemia [27]. Interestingly MPST was also identified as one of the associated genes with the development of therapy-related myeloid leukemia (t-ML) after ALL treatment among 228 B-lineage patients with almost 3-fold increased expression in comparison to the population without the t-ML complication [79]. The information about leukemic cell lines used to determine MPST expression is presented in Table 3.

CAT activity was reported to be upregulated after lysine acetylation [57]. CAT expression was upregulated in reported leukemia research [80]. These reports quite well correspond with our findings, summarized as possible elevated expression of CAT:MPST axis in T-cell ALL. The information about leukemic cell line used to determine CAT expression is presented in Table 4.

**Table 3:** Reported changes in MPST expression in different leukemia subtypes.

Study	Model	Control	Malignancy Type	MPST Conclusions	Ref.
Jurkowska et al., 2022	REH	-	B-ALL	Protein levels of MPST corresponded with mRNA level	[27]
Jurkowska et al., 2022	DND-41; MOLT-4	-	T-ALL	Protein levels of MPST corresponded with mRNA level	[27]
Jurkowska et al., 2022	MV4-11; MOLM-14	-	AML	Protein levels of MPST corresponded with mRNA level	[27]
Jurkowska et al., 2022	K562	-	CML	Protein levels of MPST corresponded with mRNA level	[27]
Liu H. et al., 2023	MT2/HeLa	-	T-cell leukemia	No change in expression of MPST over time	[81]
Wang et al., 2021	K562	CD34^+^ cells	CML	Protein levels of MPST corresponded with mRNA level, no significant change	[78]

### 3.3. Cystathionine Gamma-Lyase in Various Types of Leukemia

The CTH gene on human chromosome 1p31.1 encodes cystathionine gamma-lyase, a PLP-dependent enzyme essential for metabolizing cystathionine into cysteine, α-ketobutyrate, and ammonia. This reaction is pivotal for the liver’s production of GSH or taurine from L-cysteine [83]. Furthermore, CTH is implicated in producing H_2_S from various substrates, including homocysteine and L-cysteine, which, in turn, influences CTH expression and its biochemical activity in a concentration-dependent manner [84]. Moreover, its activity can be modified by S-nitrosylation and polysulfidation [85,86]. The enzyme’s structure is tetrameric, with each monomer hosting a PLP cofactor and 393 amino acids, divided into three distinct regions, including a PLP-binding domain that houses catalytically crucial residues [83].

The study conducted by Lazarus et al. [87] was one of the pioneering works investigating CTH activity. They observed that the CCRF-SB-cells (human B-cell ALL line) could grow on a medium supplemented with cystathionine, a cysteine precursor. In contrast, the H-SB2 cells (human T-cell ALL line) only grew when L-cystine itself was present in the medium. This led to the discovery that H-SB2 cells completely lacked active CTH enzyme. On the other hand, CCRF-SB-cells exhibited CTH activity of 0.012–0.014 µmole/h/mg protein [87]. Subsequent studies on various leukemic cell lines of ALL confirmed a decrease in CTH activity, as indicated in Table 5. Cell lines such as CEM, LAZ 2, SB2, PJT, P388, and L-1210 were identified as cysteine auxotrophs since they were unable to successfully grow in a medium lacking cystine and relying only on cystathionine. Notably, a significant decrease in CTH activity was observed in both human and murine ALL cell lines. Furthermore, both B-cell and T-cell ALL cell lines exhibited minimal residual activity of the enzyme. Iglehart et al. [88] focused their research exclusively on human cell lines to explain this phenomenon. They ruled out several potential explanations, including the presence of a substance that inhibits enzyme activity produced by leukemic cells, differences in enzyme activity caused by the purification process, differences in optimal conditions between healthy and leukemic cells that significantly affect enzyme activity, or potential impairment of cystathionine endocytosis due to transport issues. Instead, they proposed that the observed differences in enzyme activity were due to reduced intracellular levels of the enzyme. Research has further elucidated the aberrations in CTH presence and activity within leukemic cells. Glode et al. [89] revealed that two human Acute Lymphoblastic Leukemia cell lines, CEM and LAZ221, exhibit a marked decrease in CTH compared to other leukemic cell lines. Subsequent investigations by Glode [90] confirmed that both CEM and LAZ221 cell lines are L-cysteine auxotrophs (Cys^−^) and that these Cys^−^ lines showed a deficiency in active enzyme. Consequently, the heightened demand for cysteine in these leukemic Cys^−^ cell lines is presumably attributable to a decrease in intracellular cystathionase protein levels [89,90]. Kriegler et al. [91] demonstrated that it was associated with the decrease in CTH mRNA within the cells suggesting the disruption in the CTH gene expression.

Peripheral blood mononuclear cells of patients with Acute Lymphoblastic Leukemia exhibited higher cystathionine gamma-lyase protein levels and an elevated level of H_2_S, which was subsequently reduced by chemotherapy. The authors of this study demonstrated a consistent change between CTH protein and H_2_S levels [92].

CTH expression was obtained in different leukemia cell lines, including REH, DND-41, MOLT-4 (ALL cell lines), MV4-11, MOLM-14 (AML cell line), and K562 (CML cell line) [27]. It was examined that the expression of CTH was the highest in MOLM-14 and K562 cells but low in REH cells [27].

The changes in CTH expression/activity in ALL and CLL cells as well as in AML and CML cells are shown in Table 5 and Table 6, respectively.

**Table 5:** Reported changes in CTH expression/activity in ALL and CLL cells.

Study	Model	Control	Malignancy Type	CTH Conclusions	Ref.
Glode et al., 1981	NC-37, SB, Laz007, ERIC, LB-3	-	Human ALL	Cys ^+^ cells with CTH protein content. Limited growth in cystathionine supplemented medium	[90]
Iglehart et al., 1977 Glode et al., 1981	LAZ 221	-	Human ALL	Cys ^−^ Not detectable CTH protein content. Inhibited growth in supplemented medium	[88,89]
Livingston et al., 1976 Glode et al., 1981 Iglehart et al., 1977	CEM, SB2	-	Human T-ALL	Limited growth in cystathionine medium, decreased CTH activity, expressed as nmole/mg protein/h	[88,89,93]
Iglehart et al., 1977	PJT	-	Human B-ALL	Limited growth in cystathionine Medium decreased CTH activity, expressed as nmole of α-ketobutyrate formed/mg protein/h	[88]
Jurkowska et al., 2022	MOLT-4	-	Human T-ALL	Lower CTH expression than in DND-41 cells	[27]
Jurkowska et al., 2022	REH	-	Human B-ALL	Low expression of CTH	[27]
Jurkowska et al., 2022	DND-41	-	Human T-ALL	The highest expression from ALL cells	[27]
Lazarus et al., 1974	CCRF-SB, H-SB2	-	Human T-ALL	CCRF cell contains 12–14 units per gram of protein per hr of cystathionase activity. Limited activity of CTH was found in H-SB2 cells in any cells concentration	[87]
Livingston et al., 1976	P388	-	Mouse Pre-B-ALL/pre-B lymphoma	Limited growth in cystathionine medium, decreased CTH activity, expressed as nmole/mg protein/h	[93]
Livingston et al., 1976	L-1210	-	Mouse B-ALL	Limited growth in cystathionine medium, decreased CTH activity, expressed as nmole/mg protein/h	[93]
Livingston et al., 1976	P1534	-	Mouse CLL	Limited growth in cystathionine medium, decreased CTH activity, expressed as nmole/mg protein/h	[93]
Liu H. et al., 2023	MT2/HeLa	-	T-cell leukemia	Higher CTH expression after 48 h of coculture	[81]

### 3.4. Cystathionine β-Synthase in Various Types of Leukemia

Cystathionine β-synthase, a cytosolic enzyme encoded by the CBS gene located on chromosome 21q22.3, plays a crucial role in the transsulfuration pathway, catalyzing the condensation of homocysteine with serine to form cystathionine. This pyridoxal phosphate-dependent enzyme is not only instrumental in the production of H_2_S from L-cysteine but also produces by-products such as cystathionine, serine, and lanthionine [95]. The significance of CBS extends to its contribution to controlling redox homeostasis, regulating mitochondrial bioenergetics, and modulating cellular modifications involving proteins and DNA [95].

The CBS enzyme, crucial for cellular metabolism, has a 63 kDa molecular weight and is structured into 551 amino acids [95]. It features a complex architecture with an N-terminal heme domain for cofactor binding, a central catalytic domain that interacts with pyridoxal 5′-phosphate, and a C-terminal domain that responds to S-adenosylmethionine for allosteric activation. Forming a tetramer, CBS aligns with the fold II family of PLP-dependent enzymes, playing a key role in homocysteine removal via the transsulfuration pathway. Its regulation involves the C-terminal domain’s inhibition being overturned by SAM, leading to enzyme activation. This process is essential to maintaining homocysteine levels, with the enzyme’s activity modulated by its heme group, possibly acting as a redox sensor, particularly under oxidizing conditions [96,97,98]. Methylation seems to be critical modification for CBS promotor region and thus expression [95]. Under stress condition (hypoxia), CBS can undergo further modification such as S-glutathionylation, S-nitrosylation or undergo transfer to mitochondria [85,98,99]. Additionally, CBS expression is subject to hormonal regulation, highlighting its dynamic role in cellular metabolism [95].

The relevance of CBS in the context of human diseases is increasingly recognized, particularly its association with an elevated risk of leukemia in children with Down Syndrome (DS) and its correlation with atherosclerosis in DS patients. The overexpression of CBS and upregulation of its transcripts in patients with DS and AML compared to those without these conditions underscore the enzyme’s potential impact on disease pathogenesis [100,101].

Intriguingly, several complex factors influence the function of CBS in cancer. One example is heat shock factor 1 (HSF1), which plays a critical role in regulating the conversion of homocysteine to cystathionine by directly affecting CBS levels. Specifically, HSF1 binds to the CBS gene, leading to increased mRNA levels and ultimately higher CBS protein production. Targeting CBS effectively hinders prostate cancer growth and induces tumor cell death. Moreover, simultaneous inhibition of HSF1 and CBS significantly enhances the suppression of prostate cancer cell proliferation and reduces transsulfuration pathway metabolites, underscoring a potent strategy for tackling prostate cancer progression [102]. Further understanding the metabolic roles of CBS and its metabolites, homocysteine, and H_2_S, is crucial for unraveling cancer biology, offering insights into cancer pathogenesis, and enhancing therapeutic strategies [96,103].

#### 3.4.1. Cystathionine β-Synthase in Acute Myeloid Leukemia

In our previous research [27], we showed that CBS expression in AML cells was higher in MOLM-14 and lower in MV4 cell lines. The main finding was that CBS is presumably a crucial enzyme in H_2_S production in some leukemic cell lines and the role of CBS should be further examined. Information from other studies regarding CBS in AML is somewhat limited and focuses primarily on investigating the efficacy of anticancer chemotherapy in patients with increased enzyme expression, particularly due to trisomy of chromosome 21, where the gene locus is located.

A 2005 study [104] on homocysteine junction enzyme expression across various leukemia types established CBS expression levels, noting in the AML HL-60 TB line, CBS was not upregulated, with protein levels closely matching mRNA levels [104].

Children with Down Syndrome face a higher risk of developing Acute Myeloid Leukemia. They have an extremely high event-free survival rate and also a lower relapse rate. Current research explores these correlations using in vitro cell cultures and investigates chemotherapy drug modifications to enhance their efficacy, particularly through understanding the mechanisms of ara-C and daunorubicin [105,106].

Cystathionine-β-synthase may unlock specific insights into chemotherapy drug sensitivity in leukemia treatments. A study has [105] observed higher event-free survival between Down Syndrome and non-Down Syndrome AML cells treated with cytosine arabinoside (ara-C, non-active), demonstrating DS AML cells sensitivity to ara-C [105,106]. Elevated expression of CBS leads to a decrease in the dCTP pool (endogenous), thereby converting ara-C into ara-CTP (most active). Once generated, ara-CTP initiates multiple downstream biochemical pathways [105].

CBS expression was also investigated in CMK megakaryocytic leukemia (AMkL) cell line, with the non-Down Syndrome CMS cell line serving as a control. The injection of the CBS-1b luciferase gene construct into cells facilitated the observation of a 40-fold increase in activity in the AMkL line. CBS regulating role was shown to be dependent on Sp1/Sp3 binding. This finding underscores the significance of the CBS mechanism in developing AMkL chemotherapy strategies [107].

The changes in CBS expression in various AML cell lines are detailed in Table 7.

#### 3.4.2. Cystathionine β-Synthase in Chronic Myeloid Leukemia

Many leukemias, including CML, exhibit higher growth rates in environments with elevated CBS levels. Increased endogenous H_2_S levels, regulated by CBS, have been closely linked to tumorigenesis. Elevated H_2_S levels, alongside increased CBS expression, have been identified in numerous cancers. H_2_S is known to promote angiogenesis, stimulate bioenergy metabolism, and inhibit specific phosphatases. Intriguingly, these elevations in H_2_S and CBS were also observed in CML cells from the bone marrow of pediatric patients, as well as in CML-derived K562 cell lines. The measurement of CBS and H_2_S levels in K562 cells, a CML cell type, revealing upregulation that contributes to increased proliferation of primary bone marrow mononuclear cells [100] (Table 8). Inhibiting tumor growth was achieved through CBS gene knockout and the application of aminooxyacetic acid (AOAA), a broad inhibitor of PLP-dependent enzymes, on CML-derived K562 cells, which led to the activation of the apoptosis pathway. It is important to note that AOAA is also known for its ability to inhibit other critical enzymes involved in tumor progression, such as CTH and GOT, which could significantly complicate the interpretation of effects attributed solely to CBS inhibition [78,108].

Earlier studies on homocysteine metabolism and CBS expression in the K562 cell line did not explicitly reveal an upregulation of CBS [104]. However, our previous study [27], showed that the K562 cells exhibit the highest CBS expression levels of CBS among the leukemic cell lines studied. CBS level was found to correlate with different disease stages of CML and downregulation of CBS can reduce cell proliferation and induce apoptosis [78].

Increased CBS gene expression in the K562 leukemia cell line affects the activity of various drugs. The sensitivity of cytosine arabinoside in Down Syndrome myeloblasts is influenced by CBS activity. CBS enhances ara-C sensitivity through the cystathionine pathway, involving serine and homocysteine. RT-PCR measurements showed that CBS and superoxide dismutase (SOD) transcript levels are up to three times higher in DS myeloblasts compared to non-DS myeloblasts. Increased SOD expression may enhance the sensitivity to ara-C and daunorubicin in vitro, suggesting that changes in CBS expression due to other substances could impact AML progression and warrant further study [100].

CBS is responsible for the overproduction of H_2_S and the produced H_2_S acts as a metabolic suppressor in DS cells [109]. Panagaki et al. [110] reported that an increased level of CBS-derived H_2_S caused inhibition of Down Syndrome fibroblast (CRL-84) cell line proliferation compared to healthy fibroblast (CCL-110) cell line. Additionally, the activity of complex IV was lower, and electron transport in mitochondria was suppressed as was ATP formation [110].

The changes in CBS expression in various CML cell lines are shown in Table 8.

**Table 8:** CBS expression in various CML cell lines.

Study	Model	Control	Malignancy Type	CBS Conclusions	Ref.
Jurkowska et al., 2022	K562	-	Human CML	High expression of CBS	[27]
Liu H. et al., 2023	MT2/HeLa	T-cell leukemia	-	Lower CBS expression after 48 h coculture	[81]
Panagaki et al., 2019	CRL-84	CCL-110	Chronic Myeloid Leukemia at Blast Crisis	Increased level of CBS-derived H_2_S	[110]
Taub et al., 1999	DS K562	non-DS TMD cells	Human myeloblasts CML	Higher expression of CBS and SOD in DS K562 cells	[100]
Wang et al., 2021	K562	CD34+ cells	Human CML	Increased expression of CBS; CBS gene knockout inhibits CML growth	[78]
Zhang et al., 2005	K562	non-tumorigenic HepG2 cells	Human CML	No upregulation of CBS was observed	[104]

#### 3.4.3. Cystathionine β-Synthase in Acute Lymphoblastic Leukemia

Recent in vitro studies [111] showed that Acute Lymphoblastic Leukemia Down Syndrome cells respond to a broader range of chemotherapy drugs than non-DS leukemia cells. Among these drugs, methotrexate (MTX), an antifolate known for its effectiveness in high doses against ALL, was examined. The research involved the analysis of 13 cell lines, focusing on the expression of genes related to glutathione metabolism. Additionally, a thioredoxin inhibitor was used as an antioxidant within the study. The findings [111] revealed that GSH levels were correlated with MTX modulation, and exhibited resistance to inhibition, a result further supported by Gene Set Enrichment Analysis (GSEA) for B-cell precursor Acute Lymphoblastic Leukemia (BCP-ALL) and T-ALL cell lines. Moreover, GSH-related genes, specifically, gamma-glutamyltransferase 1 (*GGT1*) and thioredoxin reductase 3 (*TXNRD3*) along with *CBS* exhibited higher expression levels [111].

Contrary to findings in AML and CML cell lines, CBS expression was not detected in ALL MOLT-4 line, used in experiments [104].

Investigations into CBS expression also covered DND-41 and MOLT-4 lines [112], both classified as ALL types. The analysis revealed consistently low CBS expression across all examined ALL lines, underscoring the unexpected nature of these findings and highlighting the necessity for further research on CBS role in ALL. Notably, reactive oxygen species (ROS) levels are elevated in T-ALL cell lines compared to non-leukemic cells [112].

Our research [27], identified that CBS expression was higher in various types of leukemia compared to ALL cell lines; with notably lower expression in REH and DND-41 cells (ALL cell lines).

The changes in CBS expression in various ALL cell lines are shown in Table 9.

## 4. L-Cysteine Metabolism in Leukemia

L-cysteine appears to be essential for the proper functioning of leukemic cells both for providing antioxidative defense and for protein synthesis critical in cell division. Several studies suggest that reducing intracellular L-cysteine levels, either through inhibition of the solute carrier family 7 member 11 (SLC7A11) gene which encodes the cystine/glutamate transporter (xCT), or by directly inhibiting the xCT protein itself, may offer therapeutic benefits against malignancies [114,115,116]. System xCT, a transmembrane cystine-glutamate antiporter, imports extracellular L-cystine in exchange for glutamate. Cystine is further converted into L-cysteine inside the cell due to intracellular reducing potential. This uptake is essential as it represents the rate-limiting step for cysteine availability, critical for glutathione synthesis, a key antioxidant that maintains cellular redox balance. Inhibition of xCT, as seen with erastin, leads to GSH depletion and triggers ferroptosis, a form of cell death characterized by extensive lipid peroxidation and driven by iron-mediated Fenton reaction [117]. Some cancer cells, reliant on system x_c_^−^ for cystine import due to an inability to use the transsulfuration pathway, show heightened sensitivity to erastin-induced ferroptosis [117].

Although it is generally assumed that the primary source of L-cysteine is extracellular, a smaller amount may also be synthesized within the cells. In addition to system x_c_^−^, cells can acquire cysteine through the reverse transsulfuration pathway, converting methionine via homocysteine to cysteine, with enzymes CBS and CTH playing key roles. Consequently, the reduction in L-cysteine levels in leukemic cells could also be achieved by modulating the activity of these enzymes [117].

Cysteine, traditionally considered non-essential due to in-body synthesis from methionine, becomes essential for certain cancers, including leukemias and lymphomas, that lack the ability to synthesize it. These cancers depend on extracellular cystine/cysteine for growth, highlighting a vulnerability exploited by removing cystine or its uptake enhancers from the culture medium [118,119]. Given the low plasma concentrations of cysteine (10–20 mM cysteine vs. 100–200 mM half-cystine), lymphoid cells without endogenous cysteine synthesis depend on extracellular cysteine, often supplied by somatic cells through xCT-mediated cystine uptake [118]. While cysteine is easily absorbed by normal cells via systems like the ASC transport system, cystine transporters are less commonly found across different cell types. However, somatic cells such as fibroblasts, activated macrophages, and dendritic cells, express the xCT transporter. These cells uptake cystine from the extracellular space, convert it to cysteine internally, and then secrete cysteine back into the extracellular space, effectively completing the amino acid’s redox cycle. Tumor-associated macrophages and stromal cells thus may also promote cancer growth and therapy resistance by secreting cysteine [118,120].

Interestingly the antiporter also plays an important role in normal immune cells. Antigen-presenting cells like activated macrophages and dendritic cells express the xCT transporter, enabling the uptake of cystine from the environment, its reduction to cysteine, and subsequent secretion to support lymphocyte expansion. This cysteine provision is crucial for lymphocyte functions and growth [118]. Additionally somatic cells that support growth by secreting cysteine can be substituted with 2-mercaptoethanol (50–100 mM), a compound facilitating the cellular uptake of cystine through the L transport system as a cysteine-2-mercaptoethanol disulfide complex. Once internalized, this complex is separated into cysteine and 2-mercaptoethanol, with the latter then exiting the cell. This mechanism allows 2-mercaptoethanol to serve as a shuttle for cystine into cells, bypassing the x_c_^−^ transporter [118].

Interesting findings on this subject date back to the 1950s [121]. Research from this era demonstrated that removing cystine from the diet of mice treated with methylcholanthrene (a potent carcinogen) conferred a degree of protection against leukemia development (with incidence dropping from 92.1% in a control group to 55% with the intervention). Limiting other essential amino acids such as tryptophan and lysine did not affect leukemia incidence between the action and control groups. Furthermore the protective effect of excluding cystine from the diet was nullified after additional methionine supplementation [122,123,124,125]. Recognizing cystine as crucial for leukemic cell function led to subsequent clinical applications. Wiesberger [121] treated eight terminally ill leukemic patients, unresponsive to standard chemotherapy, with selenocystine (diseleno-dialanine)-a potent cystine influx inhibitor. The therapy was found effective in combination with standard chemotherapeutics in five of the eight patients—two with acute leukemia, two with CML, and one with “blastic CML”. Selenocystine, administered orally at a dose of 100 mg/day, was associated with severe side effects, especially nausea and vomiting. These side effects prevented therapy from extending beyond 3 weeks and even necessitated premature cessation in some cases. Despite these challenges, there were notable successes, including acute leukemia patients that obtained a 75,000/mm^3^ drop in the WBC count in 24 h, where the standard drop-in treated patients was on the level of 100,000/mm^3^ per 7 days. Another success was the effective use of 6-mercaptopurine after 21-day selenocystine therapy in a 13-year-old boy with acute leukemia (presumably ALL) resulting in satisfactory remission, despite that he had previously failed to respond for this chemotherapeutic. It was hypothesized that the lack of response in three non-responsive cases out of eight patients could be caused by the significantly shorter treatment duration. There remains a need for more specific substances causing less clinical and patient-important side effects. The precise mechanism underlying these promising results remains uncertain, as the studies did not delve into enzyme research [121].

## 5. Discussion

The reasons behind the altered expression of H_2_S-metabolizing enzymes in immunological pathologies remain unresolved. As previously noted, in normal leukocytes, the expression of these enzymes might be influenced by modulatory external triggers, such as factors impacting NF-kB expression. In pathological conditions, different factors may contribute to this altered expression. For instance, enzyme genes can be overexpressed due to pathological proteins or transcription factors produced by specific leukemic cells. As Khoury et al. [20] mentioned, many biological characteristics of tumor cells encompass gene fusions, rearrangements, and mutations. Fusions play a crucial role in the classification of various types and subtypes of diseases, especially when the identification of both involved genes is necessary or desirable for diagnosis. The term ‘rearrangements’ describes a broad spectrum of structural genomic changes that can lead to gene fusions. This term is particularly relevant when there are multiple potential fusion partners for a biologically significant gene, like KMT2A. However, it is more accurately applied to genes comprising different segments, such as immunoglobulin and T-cell receptor genes. Efforts are focused on classifying tumors based on key genetic abnormalities wherever possible.

Mutations in the genes encoding these enzymes seem unlikely, as no significant mutations or mutation frequencies have been found in existing databases. However, a more intriguing possibility is increased expression due to chromosomal abnormalities. A notable example is the Philadelphia chromosome, formed from a translocation between chromosomes 9 and 22, creating the BCR-ABL gene rearrangement. This is particularly important because, in the translocated fragment, the break occurs in the q11 region of chromosome 22. Therefore, the loci for both TST and MPST genes are located on this fragment (region 22q12.3 for both genes).

The potential impact of this gene rearrangement is difficult to evaluate, but it appears to be a very interesting area of study. It is especially worth investigating under laboratory conditions, particularly in aneuploid cell lines with more than two Philadelphia chromosomes.

Gene rearrangement can have a more global character on the chromosome and, along with genes responsible for the disease process, can significantly affect other genes by changing their expression pattern, regulation, or even creating a fusion of these genes. In the case of enzymes involved in L-cysteine metabolism, thanks to the Leukemia Database (http://bioinfo.life.hust.edu.cn/LeukemiaDB#!/) [126], a comprehensive human leukemia transcriptome database, it was possible to demonstrate the existence of numerous gene fusions encompassing all the enzymes discussed in the article (Table 10).

The fact that the fusion of these enzymes affects their function, and thus the expression and activity of the enzymes, becomes interesting. Intriguingly, different examples of fusion were found in a single cell line. For example, in K562 lines, fusions of TST_CHAF1B, ACTG1_TST, AIRE_CBS, CTH_ZNF519, MPST_TMEM212 were found, thus affecting all types of enzymes involved in L-cysteine metabolism. K562 is a CML cell line that lacks the Philadelphia chromosome and exhibits the highest expression of all the H_2_S-metabolizing enzymes. Investigating the underlying reasons for this elevated expression in K562 cells would provide valuable insights into sulfur metabolism. It is crucial to include the examination of the genetic rearrangements in these cells to understand the mechanisms driving this high enzyme expression. However, these observations come from different samples, and it is unknown whether it would be possible to detect all mentioned gene fusions in a specific cell line. The interpretation is further complicated by the fact that K562 cells are triploid, meaning they have more genetic material and, despite disturbances caused by gene fusion, may also have properly functioning sulfur metabolism genes. An interesting observation based on the gene fusions contained in the Leukemia database is that among the enzymes involved in L-cysteine metabolism, MPST was the most affected [126]. This suggests that MPST may even play a role in the pathophysiology of certain types of leukemia. The search for new fusions and gene rearrangements involving H_2_S-metabolizing enzymes and their impact on its functionality is highly needed, and further research in this area is indicated.

## 6. Conclusions

The expression of sulfurtransferases and cystathionine beta-synthase is present in normal leukocytes but varies depending on the type of immune cells, their activation state, or cell cycle stage. MPST expression is generally higher than TST across most human leukocyte populations. Both MPST and TST have very high expression levels in dendritic cells and monocytes. Neutrophils show variable TST and MPST expression, possibly due to oxidative protection needs. The expression of these enzymes can be regulated by the inflammatory factors, and the resulting H_2_S production may be a significant mediator in immune system cells. Thiosulfate sulfurtransferase shows limited expression and activity in CD4^+^ and CD8^+^ lymphocytes and some ALL cell lines, with the TST gene identified as a valuable target for distinguishing acute leukemias. The expression of MPST is altered in some leukemia cell lines, with enzyme expression potentially correlated with increased proliferative activity. CTH activity in immune system cells is relatively low and further impaired in some leukemia cell lines. This is directly linked to disruptions in CTH gene expression, potentially causing leukemia cells to become cysteine auxotrophs. CBS, essential for hydrogen sulfide production and redox regulation, is crucial in leukemia pathophysiology. Overexpression in Down Syndrome increases the risk of Acute Myeloid Leukemia, indicating a significant link between CBS activity and leukemia. Elevated CBS expression in certain leukemia types, especially Down Syndrome-related AML, enhances sensitivity to specific chemotherapy drugs. In Chronic Myeloid Leukemia, high CBS levels are associated with increased cell proliferation, suggesting CBS as a potential therapeutic target. A common feature in leukemia cells is the dysregulation of antioxidant proteins, affecting the oxidative environment and influencing cancer cell proliferation. Tumors, including leukemia cells, often rely on L-cysteine uptake for producing L-cysteine-based antioxidants. Disrupting this uptake can lead to an increase in reactive oxygen species and a decrease in cell viability. Therefore, strategies targeting the disruption of GSH, or L-cysteine metabolism could be promising in leukemia treatment, as they might overcome the protective effects these molecules provide to cancer cells. The genes encoding sulfurtransferases and cystathionine beta-synthase could undergo rearrangements and form fusion genes in leukemia cells. However, the impact of these rearrangements on the expression pattern and enzymatic activity of these genes remains unclear.

## Figures and Tables

**Table 1:** Normalized expression values of *Tst*, *Mpst*, *Cbs*, and *Cth* in various leukocytes from murine (C57Bl/6J) mice were obtained from ImmMicroarray analysis. RMA (Robust Multi-array Average) normalized values ranges are defined as follows: 0 to 5 indicates trace expression, 5 to 20 indicates very low expression, 20 to 80 indicates low expression, and 80 to 800 indicates medium expression. Each cell population is sorted according to the given phenotype. Data were obtained and adapted from the Immunological Genome Project (https://www.immgen.org/) accessed in May 2024, ProbeSet ID: 10430319 [29], and are based on [33]. Abbreviations: IgM—Immunoglobulin M, CD—Cluster of Differentiation, IL7R—Interleukin-7 Receptor, AA4.1—alanine aminotransferase 4.1, Fr—fraction, HSA—Heat-stable antigen, GL7—germinal center marker, PNA—peanut agglutinin, B220—mouse B-cell marker, DC—dendritic cell, CD11c—integrin alpha X, CD8a—CD8 alpha chain, CD4—CD4 antigen, CD11b—integrin alpha M, B220—B-cell marker, Gr1—granulocyte marker 1, MHCII—major histocompatibility complex class II, F4/80—mouse macrophage marker, SiglecF—sialic acid-binding immunoglobulin-type lectin F, HLA.DR—human leukocyte antigen-DR isotype, Ly-6C—lymphocyte antigen 6 complex, GN.BM—granulocytes from bone marrow, NK1.1—natural killer cell antigen 1.1, TCR—T-cell receptor, FSC—forward scatter, 62L—L-selectin, FoxP3—forkhead box P3, GFP—green fluorescent protein, P.I.—propidium iodide, TCRgd—gamma delta T-cell receptor, i-IEL—intraepithelial lymphocyte, and Vg5—variable region of gamma chain 5.

Population	Full Name	Immunophenotype	*Tst*	*Mpst*	*Cbs*	*Cth*
Stem cells	Long-term repopulating hematopoietic stem cell	IgM− CD24− CD117+ IL7R− CD150− CD48− AA4.1+ CD43+	146.082	106.804	84.603	36.7708
	Short-term repopulating hematopoietic stem cell	CD117+ IL7R− CD150− CD48− AA4.1+ CD43+	145.276	121.808	70.8491	33.3368
B-cells	Fr. B/C (Pro-B)	AA4+ IgM− CD19+ CD43+ HSA+	128.522	129.027	82.372	70.5058
	Fr D (Pre-B)	AA4+ IgM− CD19+ CD43− HSA+	76.1117	115.344	68.0774	51.5071
	Spleen follicular B-cells	CD19+ CD45R+ IgM+ AA4.1− CD23+ CD43− CD5−	80.4751	88.7566	73.1567	37.3882
	Germinal center B-cells, spleen	CD19+ IgM+ IgD− GL7+ PNA+	86.2635	118.769	71.1554	86.9586
	MZ (marginal zone)	CD19+ B220+ IgM++ AA4− CD23− CD21/35++	83.5685	109.307	78.2076	43.1693
	B-1a, peritoneal cavity	CD19+ B220+ IgM++ AA4− CD23− CD43+ CD5+	85.9958	114.007	76.2055	41.586
DC	Thymus CD8 DC	CD11c+ CD8a+ CD4− CD11b−	66.6965	129.121	64.9447	33.8579
	Spleen CD4+ DC	CD11c+ CD8a− CD4+ CD11b+	78.2452	86.9806	82.9736	39.9192
	Spleen CD8+ DC	CD11c+ CD8a+ CD4− CD11b−	83.6712	106.902	87.9331	40.6823
	Spleen CD8+ Plasmacytoid DC	CD11cint CD8a+ CD4+ B220+ Gr1+	74.355	89.0792	66.1802	34.3651
	Spleen CD8− Plasmacytoid DC	CD11cint CD8a− CD4+ B220+ Gr1+	74.2516	95.0246	72.6959	32.9813
	Epidermal/Langerhans DC	CD45+ MHCII+ CD11c+ CD11b+	148.269	103.714	143.884	69.008
Macrophages	Red Pulp MF	F4/80hi CD11blo CD11c− autofluorescent	92.8541	89.9649	83.3684	39.1413
	Peritoneal MF thio-elicited day 5	CD115+ MHCII+ F480lo SiglecF− CD11c+	319.041	208.446	81.3074	31.9086
Monocytes	Classical Mo, MHCII-	CD115+ B220− Ly−6C+ MHCII−	79.1397	86.1923	72.2166	35.7548
	Classical Mo, MHCII+	CD115+ B220− Ly−6C+ to int MHCII+	83.0767	91.1758	87.5789	32.5929
	Classical Mo, MHCII−	CD115+ B220− Ly−6C+ MHCII− F480int CD11c− CD11bhi	81.6857	95.6535	83.2502	34.5642
GN.BM	Neutrophils from bone marrow	CD11b+ Gr1+ 7/4hi	599.476	209.029	83.9213	36.2583
NK-cells	Splenic NK-cells	CD3− NK1.1+	90.9751	114.136	118.534	43.4223
	Splenic NK-cells, Ly49CI− subset	CD3− NK1.1+ Ly49C/I−	78.4708	104.925	96.7076	37.6034
	Splenic NK-cells, Ly49CI+ subset	CD3− NK1.1+ Ly49C/I+	89.138	105.731	99.2288	33.6786
	Splenic NK-cells, day 1 post-MCMV	CD3− NK1.1+	90.8826	90.4106	92.8953	36.5564
T-cells	Double-positive, small resting	4+ 8+ TCR-/lo FSClo	100.32	128.988	95.2632	39.1057
	CD4 single-positive, mature	4+ 8− TCRhi 24-/lo	90.0542	136.763	77.6441	35.2188
	CD8 single-positive, mature	4− 8+ TCRhi 24-/lo	85.2996	135.466	79.262	33.4188
	Spleen Naive CD4	CD3e+ 4+ 8− 19− 62Lhi	83.231	115.368	79.2118	33.3387
	Spleen memory-phenotype CD4	4+ 8− TCR+ 25− 44hi 122lo	95.8972	126.664	96.9553	40.6268
	Peyers patches naive CD4	4+ 8− TCR+ 25− 62Lhi 44lo	71.6536	97.4748	96.5298	40.8235
	SplCD25+Tregs	GFP-FoxP3 knockin mice sorted on 4+ 8− GFP+ 25+	99.9732	126.037	106.113	42.9116
	Spleen naive CD8	4− 8+ TCR+ 25− 62Lhi 44lo	98.4326	127.671	106.113	40.9713
	Subcutaneous LN Memory-phenotype CD8	4− 8+ TCR+ 25− 44hi 122hi	97.2038	130.049	113.793	39.1141
	Spleen OT1 tg naive CD8	CD8+ CD45.1+ B220− CD45.2− Gr1− NK1.1− CD4− MHCII− P.I.−	77.9145	117.015	67.7647	32.1435
	Spleen OT1 tg effectors CD8 6 days after vesicular stomatitis virus infection	CD8+ CD45.1+ B220− CD45.2− Gr1− NK1.1− CD4− MHCII− P.I.−	92.8383	108.895	107.028	46.4714
	Spleen OT1 tg memory CD8 Day 45 days after vesicular stomatitis virus infection	CD8+ CD45.1+ B220− CD45.2− Gr1− NK1.1− CD4− MHCII− P.I.−	88.5838	100.387	90.1369	35.8652
	Spleen OT1 tg memory CD8 Day 106 days after vesicular stomatitis virus infection	CD8+ CD45.1+ B220− CD45.2− Gr1− NK1.1− CD4− MHCII− P.I.−	100.211	108.869	110.669	51.48
	Thymic TCRgd, all DN	TCRd+ CD4− CD8−	95.4432	138.617	82.7585	36.9967
	Spleen TCRgd, all DN	TCRd+ CD4− CD8−	98.5398	109.855	104.663	41.7655
	i-IEL Vg5+, activated	Vg5+ TCRd+ CD44+ CD8aa+	91.834	124.204	69.0243	32.1892

**Table 2:** The normalized expression values of TST, MPST, CBS, and CTH within human leukocytes are categorized as follows: 0 to 5 indicates trace expression, 5 to 20 indicates very low expression, 20 to 80 indicates low expression, and 80 to 800 indicates medium expression. The data were obtained from ULI RNASeq through the Immunological Genome Project (https://www.immgen.org/), accessed in May 2024 [29] and redirected from the Immune Cell Atlas (http://immunecellatlas.net/). Based on reference [33]. Abbreviations: ILC—innate lymphoid cells; abT—alpha beta T-cells; Mo—monocytes; DC—dendritic cells; PBMCs—peripheral blood mononuclear cells; CD—cluster of differentiation; HLA.DR—human leukocyte antigen-DR isotype; TCRb—T-cell receptor beta; CD19^+^—CD19 positive; IgD^+^ —immunoglobulin D positive; CD56hi—high expression of CD56; CD62Lhi—high expression of CD62L; CD161dull—dull expression of CD161; CD16lo—low expression of CD16.

Cell	Population Name	Description	TST	MPST	CBS	CTH
B-cells	B.NveIgD+27	CD19^+^ IgD^+^ CD27− PBMCs	10.7159	112.302	11.442	14.9944
	B.MemIgD-27+38	CD19^+^ IgD− CD27+ CD38− PBMCs	2.78259	40.7265	1.89768	4.58114
	B.TransIgD+27	CD19^+^ IgD+CD27− PBMCs	2.3284	55.689	1	13.1962
ILC	ILC.Nkimm.56hi16	CD19− CD56hi PBMCs	32.8905	183.559	1	1
	ILC.Nkmat.56lo16hi57	CD19− CD56mid CD16+ CD57− PBMCs	15.808	96.8911	1.72836	4.93923
	ILC.Nkmem.56lo16hi57hi	CD19− CD56mid CD16+ CD57+ PBMCs	6.80326	85.6913	1	2.32381
abT-cells	T.4Nve.CD3+4+RA+62L	DR− TCRb+ CD4+ CD8− CD127+ CD25− CD54RAhi CD62Lhi PBMCs	4.13621	6.54517	1	11.636
	T.4EffMem.CD3+4+RA-62L	DR− TCRb+ CD4+ CD8− CD127+ CD54RA− CD62L− PBMCs	10.307	39.5897	2.47339	4.54848
	T.8Nve.CD3+8+RA+62L	DR− TCRb+ CD4− CD8+ CD127+ CD25− CD54RAhi CD62Lhi PBMCs	1.77288	5.32207	1.77288	8.8763
	T.8EffMem.CD3+8+RA-62L	DR− TCRb+ CD4− CD8+ CD127+ CD54RA− CD62L− PBMCs	5.13594	84.3976	1	6.3522
	T.NKT.Va24	CD19− CD3+ XXX PBMCs	2.42901	51.0113	1	1.47634
	T.MAIT.4	CD19− CD3+ CD4+ CD8− Va72.2+ CD161dull PBMCs	7.06241	24.8644	1	7.76393
	T.MAIT.8	CD19− CD3+ CD4− CD8+ Va72.2+ CD161dull PBMCs	6.89477	80.4932	1	6.50934
	T.Treg.rest	CD19− CD4− CD25+ CD127− CD45RA+ PBMCs	2.51367	11.5957	1	15.206
	T.Treg.act	CD19− CD4− CD25+ CD127− CD45RA− PBMCs	4.71504	122.602	1	6.44366
Mo	Mo.16	HLA.DR+ CD16hi CD14lo PBMCs	19.0173	38.9905	1	4.29847
	Mo.14	HLA.DR+ CD16lo CD14hi PBMCs	84.3467	187.787	1.69173	1
DC	DC.DC1.141	HLA.DR+ CD141+ CD16− CD14− PBMCs	61.2666	249.952	1.71918	3.87303
	DC.DC5.AXL+SIGLEC6	HLA.DR+ CD141− CD16− CD14− AXL+ SIGLEC6+ PBMCs	84.6527	155.452	2.25322	1
	DC.DC6.123	HLA.DR+ CD141− CD16− CD14− AXL− SIGLEC6− CD123+ CD11c− PBMCs	6.21346	8.95739	1.27439	3.24191

**Table 4:** Reported changes in CAT expression in different leukemia subtypes.

Study	Model	Control	Malignancy Type	CAT Conclusions	Ref.
Chen et al., 2020	Nras(G12D)/MLL-AF9 mouse cells	Embryonic iMEF cells	AML	Expression of CAT mRNA was up regulated	[80]
Chen et al., 2020	MOLM13	Embryonic iMEF cells	AML	Expression of CAT mRNA was up regulated	[80]
Shen et al., 2016	KBM7	KBM7-Mu	CML	Expression of CAT mRNA facilitate imatinib resistance	[82]

**Table 6:** Reported changes in CTH expression/activity in AML and CML cells.

Study	Model	Control	Malignancy Type	CTH Conclusions	Ref.
Jurkowska et al., 2022	MOLM-14	-	Human monocytic AML	Increased CTH expression	[27]
Jurkowska et al., 2022	MV4-11	-	Human macrophages AML	Lower expression than in MOLM-14 cells	[27]
Jurkowska et al., 2022	K562	-	Human CML	Increased CTH expression	[27]
Link et al., 1983	ML1	-	Human AML	Low CTH content	[94]
Livingston et al., 1976	LW12	-	Rat AML	Limited growth in medium lacking L-cystine	[93]
Livingston et al., 1976	RBL-1	-	Rat basophilic leukemia	Limited growth in medium lacking L-cystine	[93]
Wang et al., 2021	K562	CD34^+^ cells	CML	Protein levels of CTH correspond with mRNA level, no significant change	[78]

**Table 7:** CBS expression in various AML cell lines.

Study	Model	Control	Malignancy Type	CBS Conclusions	Ref
Ge et al., 2003	DS megakaryocytic AMkL	CMS, non-DS AMkL cell line	Human megakaryoblasts AML	Higher expression of CBS in CMK line, Sp1/Sp3 controls CBS activity	[107]
Jurkowska et al., 2022	MOLM-14	-	Human monocytic AML	Higher expression than in MV4 cells	[27]
Jurkowska et al., 2022	MV4-11	-	Human macrophages AML	Lower expression than in MOLM-14 cells	[27]
Taub et al., 1996	Leukemia cells from patients with DS- AML	non-DS AML cells	M_7_-AML	Significantly higher CBS expression in DS-AML than in control cells	[106]
Taub and Ge, 2005	DS megakaryocytic AMkL	non-DS CMS cells	Human megakaryoblasts AML	CBS upregulated expression (median 12-fold higher in DS) increased MTHFR level.	[105]
Zhang et al., 2005	HL-60 TB	non-tumorigenic HepG2 cells	Human promyelocytic AML	CBS expression was not detectable	[104]

**Table 9:** CBS expression in various ALL cell lines. ↑ indicates increased expression.

Study	Model	Control	Malignancy Type	CBS and GSH Conclusions	Ref.
Canevarolo et al., 2022	REH MTX (+)	untreated cells—MTX (-)	Childhood B-ALL	↑ GSH-related genes expression incl. CBS	[111]
Canevarolo et al., 2022	RS4;11 MTX (+)	untreated cells—MTX (-)	Human B-ALL	↑ GSH-related genes expression incl. CBS	[111]
Canevarolo et al., 2022	NALM-30 MTX (+)	untreated cells—MTX (-)	Human mixed phenotype ALL	↑ GSH-related genes expression incl. CBS	[111]
Canevarolo et al., 2022	697 cell line MTX (+)	untreated cells—MTX (-)	Human B-cell in ALL relapses	↑ GSH-related genes expression incl. CBS	[111]
Canevarolo et al., 2022	ALL-SILL MTX (+)	untreated cells—MTX (-)	Childhood T-ALL	↑ GSH-related genes expression incl. CBS	[111]
Canevarolo et al., 2022	CCRF-CEM; HPB-ALL; Jurkat; MOLT-4; P12-ICHIKAWA; TALL-1 MTX (+)	untreated cells—MTX (-)	Human T-ALL	↑ GSH-related genes expression incl. CBS	[111]
Fotoohi et al., 2009	Drug resistant clones of MTX/7-OHMTX MOLT-4	parental MOLT-4	Human ALL	No detectable expression of CBS in clones resistant to 7-OHMTX, a 50% reduction in CBS expression in clones resistant to MTX	[113]
Jurkowska et al., 2022	DND-41	-	Human T-ALL	Low expression of CBS	[27]
Jurkowska et al., 2022	MOLT-4	-	Human T-ALL	Statistically significant CBS expression	[27]
Jurkowska et al., 2022	REH	-	Human B-ALL	Low expression of CBS	[27]
Wang et al., 2018	DND-41 cocultured with MSCs	non-coculture control group	Human T-ALL	Low expression of CBS	[112]
Wang et al., 2018	MOLT-4 cocultured with MSCs	non-coculture control group	Human T-ALL	Low expression of CBS	[112]
Taub et al., 1999	CCRF-CEM DS cell line	non-DS TMD cells	Human myeloblasts ALL	Higher expression of CBS	[100]
Zhang et al., 2005	MOLT-4	non-tumorigenic HepG2 cells	Human T-ALL	No changes in CBS expression	[104]

**Table 10:** The sulfurtransferases and CBS gene fusions were reported within human leukemic cells. Abbreviations: Chr1, Chr2—chromosomes of Gene1 and Gene2, respectively; S1, S2—strand 1 and strand 2, respectively. Data sourced from Leukemia DB—human leukemia transcriptome database (http://bioinfo.life.hust.edu.cn/LeukemiaDB#!/), accessed in December 2023 [126].

FusionName	GeneName1	GeneName2	Chr1	S1	Chr2	S2	InfoGene1	InfoGene2
MPST_TMEM212	MPST	TMEM212	22	+	3	+	mercaptopyruvate sulfurtransferase	transmembrane protein 212
MPST_CDK5RAP2	MPST	CDK5RAP2	22	+	9	−	mercaptopyruvate sulfurtransferase	CDK5 regulatory subunit associated protein 2
MPST_FBXO41	MPST	FBXO41	22	+	2	−	mercaptopyruvate sulfurtransferase	F-box protein 41
MPST_OCIAD2	MPST	OCIAD2	22	+	4	−	mercaptopyruvate sulfurtransferase	OCIA domain containing 2
MPST_FAM106A	MPST	FAM106A	22	+	17	−	mercaptopyruvate sulfurtransferase	family with sequence similarity 106 member A
MPST_ZNF396	MPST	ZNF396	22	+	18	−	mercaptopyruvate sulfurtransferase	zinc finger protein 396
MPST_RPAP2	MPST	RPAP2	22	+	1	+	mercaptopyruvate sulfurtransferase	RNA polymerase II associated protein 2
MPST_RPN1	MPST	RPN1	22	+	3	−	mercaptopyruvate sulfurtransferase	ribophorin I
MPST_RBM39	MPST	RBM39	22	+	20	−	mercaptopyruvate sulfurtransferase	RNA binding motif protein 39
MPST_KDELC2	MPST	KDELC2	22	+	11	−	mercaptopyruvate sulfurtransferase	KDEL motif containing 2
MPST_SUPT16H	MPST	SUPT16H	22	+	14	−	mercaptopyruvate sulfurtransferase	SPT16 homolog, facilitates chromatin remodeling subunit
MPST_TRIM52	MPST	TRIM52	22	+	5	−	mercaptopyruvate sulfurtransferase	tripartite motif containing 52
MPST_SLC43A1	MPST	SLC43A1	22	+	11	−	mercaptopyruvate sulfurtransferase	solute carrier family 43, member 1
MPST_MTRNR2L8	MPST	MTRNR2L8	22	+	11	−	mercaptopyruvate sulfurtransferase	MT-RNR2-like 8
MPST_USP8	MPST	USP8	22	+	15	+	mercaptopyruvate sulfurtransferase	ubiquitin specific peptidase 8
SGTB_MPST	SGTB	MPST	5	−	22	+	small glutamine-rich TPR-containing, beta	mercaptopyruvate sulfurtransferase
MIER1_MPST	MIER1	MPST	1	+	22	+	mesoderm induction early response 1	mercaptopyruvate sulfurtransferase
SLC10A3_MPST	SLC10A3	MPST	X	−	22	+	solute carrier family 10 member 3	mercaptopyruvate sulfurtransferase
PCDHB13_MPST	PCDHB13	MPST	5	+	22	+	protocadherin beta 13	mercaptopyruvate sulfurtransferase
SLC30A9_MPST	SLC30A9	MPST	4	+	22	+	solute carrier family 30, member 9	mercaptopyruvate sulfurtransferase
ALG11_MPST	ALG11	MPST	13	+	22	+	ALG11, alpha-1,2-mannosyltransferase	mercaptopyruvate sulfurtransferase
RAI1_MPST	RAI1	MPST	17	+	22	+	retinoic acid induced 1	mercaptopyruvate sulfurtransferase
NBAS_MPST	NBAS	MPST	2	−	22	+	neuroblastoma amplified sequence	mercaptopyruvate sulfurtransferase
CCNC_MPST	CCNC	MPST	6	−	22	+	cyclin C	mercaptopyruvate sulfurtransferase
TGM2_MPST	TGM2	MPST	20	−	22	+	transglutaminase 2	mercaptopyruvate sulfurtransferase
KCNJ11_MPST	KCNJ11	MPST	11	−	22	+	potassium channel, inwardly rectifying J11	mercaptopyruvate sulfurtransferase
ITGAE_MPST	ITGAE	MPST	17	−	22	+	integrin subunit alpha E	mercaptopyruvate sulfurtransferase
ZNF736_MPST	ZNF736	MPST	7	+	22	+	zinc finger protein 736	mercaptopyruvate sulfurtransferase
GOLGA2_MPST	GOLGA2	MPST	9	−	22	+	golgin A2	mercaptopyruvate sulfurtransferase
BLVRA_MPST	BLVRA	MPST	7	+	22	+	biliverdin reductase A	mercaptopyruvate sulfurtransferase
TST_CHAF1B	TST	CHAF1B	22	−	21	+	thiosulfate sulfurtransferase (rhodanese)	chromatin assembly factor 1 subunit B
ACTG1_TST	ACTG1	TST	17	−	22	−	actin gamma 1	thiosulfate sulfurtransferase (rhodanese)
DFFB_CTH	DFFB	CTH	1	+	1	+	DNA fragmentation factor, 40kDa, beta polypeptide (caspase-activated DNase)	cystathionine gamma-lyase
GPBP1L1_CTH	GPBP1L1	CTH	1	−	1	+	GC-rich promoter binding protein 1-like 1	cystathionine gamma-lyase
SERPINF1_CTH	SERPINF1	CTH	17	+	1	+	serpin peptidase inhibitor, clade F, member 1	cystathionine gamma-lyase
SAMD3_CTH	SAMD3	CTH	6	−	1	+	sterile alpha motif domain containing 3	cystathionine gamma-lyase
TNRC6C-AS1_CTH	TNRC6C-AS1	CTH	17	−	1	+	TNRC6C antisense RNA 1	cystathionine gamma-lyase
AIG1_CTH	AIG1	CTH	6	+	1	+	androgen-induced 1	cystathionine gamma-lyase
MYLK_CTH	MYLK	CTH	3	−	1	+	myosin light chain kinase	cystathionine gamma-lyase
SLC25A32_CTH	SLC25A32	CTH	8	−	1	+	solute carrier family 25, member 32	cystathionine gamma-lyase
ORMDL2_CTH	ORMDL2	CTH	12	+	1	+	ORMDL sphingolipid biosynthesis regulator 2	cystathionine gamma-lyase
CTH_CIZ1	CTH	CIZ1	1	+	9	−	cystathionine gamma-lyase	CDKN1A interacting zinc finger protein 1
CTH_ZNF519	CTH	ZNF519	1	+	18	−	cystathionine gamma-lyase	zinc finger protein 519
CTH_NDUFA10	CTH	NDUFA10	1	+	2	−	cystathionine gamma-lyase	NADH:ubiquinone oxidoreductase subunit A10
CTH_SLC22A11	CTH	SLC22A11	1	+	11	+	cystathionine gamma-lyase	solute carrier family 22, member 11
CBS_TMEM63A	CBS	TMEM63A	21	−	1	−	cystathionine-beta-synthase	transmembrane protein 63A
CBS_ZNF619	CBS	ZNF619	21	−	3	+	cystathionine-beta-synthase	zinc finger protein 619
CBS_LRRC8B	CBS	LRRC8B	21	−	1	+	cystathionine-beta-synthase	leucine-rich repeat containing 8 family member B
CBS_EVI5	CBS	EVI5	21	−	1	−	cystathionine-beta-synthase	ecotropic viral integration site 5
CBS_PRKX	CBS	PRKX	21	−	X	−	cystathionine-beta-synthase	protein kinase, X-linked
CBS_FDX1L	CBS	FDX1L	21	−	19	−	cystathionine-beta-synthase	ferredoxin 1-like
BS_UBXN7	CBS	UBXN7	21	−	3	−	cystathionine-beta-synthase	UBX domain protein 7
AIRE_CBS	AIRE	CBS	21	+	21	−	autoimmune regulator	cystathionine-beta-synthase
OTUD3_CBS	OTUD3	CBS	1	+	21	−	OTU deubiquitinase 3	cystathionine-beta-synthase

## Data Availability

No new data were included in this review.

## References

[1] Andrés C.M.C., Pérez de la Lastra J.M., Andrés Juan C., Plou F.J., Pérez-Lebeña E. (2023). Chemistry of hydrogen sulfide-pathological and physiological functions in mammalian cells. Cells.

[2] Kamoun P. (2004). Endogenous Production of hydrogen sulfide in mammals. Amino Acids.

[3] McBean G.J. (2012). The transsulfuration pathway: A source of cysteine for glutathione in astrocytes. Amino Acids.

[4] Berndt C., Lillig C.H. (2017). Glutathione, glutaredoxins, and iron. Antioxid. Redox Signal..

[5] Ogata F.T., Branco V., Vale F.F., Coppo L. (2021). Glutaredoxin: Discovery, redox defense and much more. Redox Biol..

[6] Rhee S.G. (2016). Overview on peroxiredoxin. Mol. Cells.

[7] Sbodio J.I., Snyder S.H., Paul B.D. (2019). Regulators of the transsulfuration pathway. Br. J. Pharmacol..

[8] Wu D., Si W., Wang M., Lv S., Ji A., Li Y. (2015). Hydrogen sulfide in cancer: Friend or foe?. Nitric Oxide-Biol. Chem..

[9] Melino S., Sabelli R., Paci M. (2011). Allyl sulfur compounds and cellular detoxification system: Effects and perspectives in cancer therapy. Amino Acids.

[10] Bonifácio V.D.B., Pereira S.A., Serpa J., Vicente J.B. (2021). Cysteine metabolic circuitries: Druggable targets in cancer. Br. J. Cancer.

[11] Hänzelmann P., Dahl J.U., Kuper J., Urban A., Müller-Theissen U., Leimkühler S., Schindelin H. (2009). Crystal structure of YnjE from Escherichia coli, a sulfurtransferase with three rhodanese domains. Protein Sci..

[12] Zhang H.F., Klein Geltink R.I., Parker S.J., Sorensen P.H. (2022). Transsulfuration, minor player or crucial for cysteine homeostasis in cancer. Trends Cell Biol..

[13] Jurkowska H., Wróbel M. (2018). Inhibition of human neuroblastoma cell proliferation by N-acetyl-L-cysteine as a result of increased sulfane sulfur level. Anticancer Res..

[14] Jurkowska H., Wróbel M., Kaczor-Kamińska M., Jasek-Gajda E. (2017). A possible mechanism of inhibition of U87MG and SH-SY5Y cancer cell proliferation by diallyl trisulfide and other aspects of its activity. Amino Acids.

[15] Jurkowska H., Uchacz T., Roberts J., Wróbel M. (2011). Potential therapeutic advantage of ribose-cysteine in the inhibition of astrocytoma cell proliferation. Amino Acids.

[16] Jurkowska H., Wróbel M. (2008). N-Acetyl-L-cysteine as a source of sulfane sulfur in astrocytoma and astrocyte cultures: Correlations with cell proliferation. Amino Acids.

[17] Majumder A. (2023). Targeting Homocysteine and Hydrogen Sulfide Balance as Future Therapeutics in Cancer Treatment. Antioxidants.

[18] Zolfaghari M., Sajedi H. (2022). A survey on automated detection and classification of acute leukemia and WBCs in microscopic blood cells. Multimed. Tools. Appl..

[19] Jin M.W., Xu S.M., An Q., Wang P. (2016). A review of risk factors for childhood leukemia. Eur. Rev. Med. Pharmacol. Sci..

[20] Khoury J.D., Solary E., Abla O., Akkari Y., Alaggio R., Apperley J.F., Bejar R., Berti E., Busque L., Chan J.K.C. (2022). The 5th edition of the World Health Organization Classification of Haematolymphoid Tumours: Myeloid and Histiocytic/Dendritic Neoplasms. Leukemia.

[21] Alaggio R., Amador C., Anagnostopoulos I., Attygalle A.D., de Oliveira Araujo I.B., Berti E., Bhagat G., Borges A.M., Boyer D., Calaminici M. (2022). International Agency for Research on Cancer/World Health Organization. Correction: The 5th edition of The World Health Organization Classification of Haematolymphoid Tumours: Lymphoid Neoplasms. Leukemia.

[22] Pulte D., Jansen L., Brenner H. (2020). Changes in long term survival after diagnosis with common hematologic malignancies in the early 21st century. Blood Cancer J..

[23] Amini L., Silbert S.K., Maude S.L., Nastoupil L.J., Ramos C.A., Brentjens R.J., Sauter C.S., Shah N.N., Abou-El-Enein M. (2022). Preparing for CAR T cell therapy: Patient selection, bridging therapies and lymphodepletion. Nat. Rev. Clin. Oncol..

[24] Osman A.E.G., Deininger M.W. (2021). Chronic Myeloid Leukemia: Modern therapies, current challenges and future directions. Blood Rev..

[25] Marvin-Peek J., Savani B.N., Olalekan O.O., Dholaria B. (2022). Challenges and advances in chimeric antigen receptor therapy for Acute Myeloid Leukemia. Cancers.

[26] Kruse A., Abdel-Azim N., Kim H.N., Ruan Y., Phan V., Ogana H., Wang W., Lee R., Gang E.J., Khazal S. (2020). Minimal residual disease detection in Acute Lymphoblastic Leukemia. IJMS.

[27] Jurkowska H., Wróbel M., Jasek-Gajda E., Rydz L. (2022). Sulfurtransferases and cystathionine beta-synthase expression in different human leukemia cell lines. Biomolecules.

[28] Wu C., Jin X., Tsueng G., Afrasiabi C., Su A.I. (2016). BioGPS: Building your own mash-up of gene annotations and expression profiles. Nucleic Acids Res..

[29] Heng T.S., Painter M.W. (2008). Immunological Genome Project Consortium. The Immunological Genome Project: Networks of gene expression in immune cells. Nat. Immunol..

[30] Mårtensson J., Sörbo B. (1978). Human β-mercaptopyruvate sulfurtransferase: Distribution in cellular compartments of blood and activity in erythrocytes from patients with hematological disorders. Clin. Chim. Acta.

[31] Valentine W.N., Frankenfeld J.K. (1974). 3-Mercaptopyruvate sulfurtransferase (EC 2.8.1.2): A simple assay adapted to human blood cells. Clin. Chim. Acta.

[32] Wróbel M., Grabowska A., Włodek L., Czubak J., Marcinkiewicz J. (2002). Sulfurtransferases activity and sulfane sulfur level in cells of the immune system. Preliminary report. Cent. Eur. J. Immunol..

[33] Dilek N., Papapetropoulos A., Toliver-Kinsky T., Szabo C. (2020). Hydrogen sulfide: An endogenous regulator of the immune system. Pharmacol. Res..

[34] Schmiedel B.J., Singh D., Madrigal A., Valdovino-Gonzalez A.G., White B.M., Zapardiel-Gonzalo J., Ha B., Altay G., Greenbaum J.A., McVicker G. (2018). Impact of genetic polymorphisms on human immune cell gene expression. Cell.

[35] Monaco G., Lee B., Xu W., Mustafah S., Hwang Y.Y., Carré C., Burdin N., Visan L., Ceccarelli M., Poidinger M. (2019). RNA-Seq signatures normalized by mrna abundance allow absolute deconvolution of human immune cell types. Cell Rep..

[36] Goldstein J.L., Campbell B.K., Gartler S.M. (1972). Cystathionine synthase activity in human lymphocytes: Induction by phytohemagglutinin. J. Clin. Investig..

[37] Allsop J., Watts R.W. (1975). Methionine adenosyltransferase, cystathionine beta-synthase and cystathionine gamma-lyase activity of rat liver subcellular particles, human blood cells and mixed white cells from rat bone marrow. Clin. Sci. Mol. Med. Suppl..

[38] Devi K.S., Devi A.R., Kondaiah P. (1998). Amplification of phenylalanine hydroxylase and cystathionine beta-synthase transcripts in human peripheral lymphocytes by RT-PCR. Biochem. Mol. Biol. Int..

[39] Katko M., Zavaczki E., Jeney V., Paragh G., Balla J., Varga Z. (2012). Homocysteine metabolism in peripheral blood mononuclear cells: Evidence for cystathionine beta-synthase activity in resting state. Amino Acids.

[40] Garg S., Vitvitsky V., Gendelman H.E., Banerjee R. (2006). Monocyte differentiation, activation, and mycobacterial killing are linked to transsulfuration-dependent redox metabolism. J. Biol. Chem..

[41] Badiei A., Chambers S.T., Gaddam R.R., Fraser R., Bhatia M. (2016). Cystathionine-gamma-lyase gene silencing with siRNA in monocytes/macrophages protects mice against acute pancreatitis. Appl. Microbiol. Biotechnol..

[42] Li L., Whiteman M., Moore P.K. (2009). Dexamethasone inhibits lipopolysaccharide-induced hydrogen sulphide biosynthesis in intact cells and in an animal model of endotoxic shock. J. Cell. Mol. Med..

[43] Ivanciuc T., Sbrana E., Casola A., Garofalo R.P. (2019). Cystathionine γ-lyase deficiency enhances airway reactivity and viral-induced disease in mice exposed to side-stream tobacco smoke. Pediatr. Res..

[44] Vuillefroy de Silly R., Coulon F., Poirier N., Jovanovic V., Brouard S., Ferchaud-Roucher V., Blancho G., Vanhove B. (2012). Transplant tolerance is associated with reduced expression of cystathionine-γ-lyase that controls IL-12 production by dendritic cells and TH-1 immune responses. Blood.

[45] Kolluru G.K., Bir S.C., Yuan S., Shen X., Pardue S., Wang R., Kevil C.G. (2015). Cystathionine γ-lyase regulates arteriogenesis through NO-dependent monocyte recruitment. Cardiovasc. Res..

[46] Park S.J., Yoon B.H., Kim S.K., Kim S.Y. (2019). GENT2: An updated gene expression database for normal and tumor tissues. BMC Med. Genom..

[47] Toohey J.I., Cooper A.J.L. (2014). Thiosulfoxide (sulfane) sulfur: New chemistry and new regulatory roles in biology. Molecules.

[48] Gal E.M., Fung F.H., Greenberg D.M. (1952). Studies on the biological action of malononitriles II. Distribution of rhodanese (transulfurase) in the tissues of normal and tumor-bearing animals and the effect of malononitriles thereon. Cancer Res..

[49] Koeffler H.P., Lowe L., Golde D.W. (1980). Amygdalin (Laetrile): Effect on clonogenic cells from human myeloid leukemia cell lines and normal human marrow. Cancer Treat. Rep..

[50] Stelmaszyńska T. (1985). Formation of HCN by human phagocytosing neutrophils--1. Chlorination of Staphylococcus epidermidis as a source of HCN. Int. J. Biochem..

[51] Stelmaszyńska T. (1986). Formation of HCN and its chlorination to C1cn by stimulated human neutrophils-2. oxidation of thiocyanate as a source of HCN. Int. J. Biochem..

[52] Dubitzky W., Scientist F.D., Berrar D. (2002). Comparing symbolic and subsymbolic machine learning approaches to classification of cancer and gene identification. Methods of Microarray Data Analysis.

[53] Sewak M.S., Reddy N.P., Duan Z.H. (2009). Gene expression based leukemia sub-classification using committee neural networks. Bioinform. Biol. Insights.

[54] Yadav P.K., Yamada K., Chiku T., Koutmos M., Banerjee R. (2013). Structure and kinetic analysis of H_2_S production by human mercaptopyruvate sulfurtransferase. J. Biol. Chem..

[55] Nagahara N., Ito T., Kitamura H., Nishino T. (1998). Tissue and subcellular distribution of mercaptopyruvate sulfurtransferase in the rat: Confocal laser fluorescence and immunoelectron microscopic studies combined with biochemical analysis. Histochem. Cell Biol..

[56] Giuffrè A., Vicente J.B. (2018). Hydrogen sulfide biochemistry and interplay with other gaseous mediators in mammalian physiology. Oxid. Med. Cell. Longev..

[57] Hipólito A., Nunes S.C., Vicente J.B., Serpa J. (2020). Cysteine aminotransferase (CAT): A pivotal sponsor in metabolic remodeling and an ally of 3-mercaptopyruvate sulfurtransferase (MST) in cancer. Molecules.

[58] Pedre B., Dick T.P. (2021). 3-Mercaptopyruvate sulfurtransferase: An enzyme at the crossroads of sulfane sulfur trafficking. Biol. Chem..

[59] Williams R.A.M., Kelly S.M., Mottram J.C., Coombs G.H. (2003). 3-Mercaptopyruvate sulfurtransferase of Leishmania contains an unusual C-terminal extension and is involved in thioredoxin and antioxidant metabolism. J. Biol. Chem..

[60] Augsburger F., Szabo C. (2020). Potential role of the 3-mercaptopyruvate sulfurtransferase (3-MST)-hydrogen sulfide (H_2_S) pathway in cancer cells. Pharmacol. Res..

[61] Nagahara N. (2018). Multiple role of 3-mercaptopyruvate sulfurtransferase: Antioxidative function, H_2_S and polysulfide production and possible SOx production. Br. J. Pharmacol..

[62] Rydz L., Wróbel M., Jurkowska H. (2021). Sulfur administration in Fe–S cluster homeostasis. Antioxidants.

[63] Giuffrè A., Tomé C.S., Fernandes D.G.F., Zuhra K., Vicente J.B. (2020). Hydrogen sulfide metabolism and signaling in the tumor microenvironment. Adv. Exp. Med. Biol..

[64] Pedre B., Talwar D., Barayeu U., Schilling D., Luzarowski M., Sokolowski M., Glatt S., Dick T.P. (2023). 3-Mercaptopyruvate sulfurtransferase is a protein persulfidase. Nat. Chem. Biol..

[65] Wakabayashi N., Dinkova-Kostova A.T., Holtzclaw W.D., Kang M.I., Kobayashi A., Yamamoto M., Kensler T.W., Talalay P. (2004). Protection against electrophile and oxidant stress by induction of the phase 2 response: Fate of cysteines of the Keap1 sensor modified by inducers. Proc. Natl. Acad. Sci. USA.

[66] Szabo C. (2021). Hydrogen sulfide, an endogenous stimulator of mitochondrial function in cancer cells. Cells.

[67] Valentine W.N., Toohey J.I., Paglia D.E., Nakatani M., Brockway R.A. (1987). Modification of erythrocyte enzyme activities by persulfides and methanethiol: Possible regulatory role. Proc. Natl. Acad. Sci. USA.

[68] Zivanovic J., Kouroussis E., Kohl J.B., Adhikari B., Bursac B., Schott-Roux S., Petrovic D., Miljkovic J.L., Thomas-Lopez D., Jung Y. (2019). Selective persulfide detection reveals evolutionarily conserved antiaging effects of S-sulfhydration. Cell Metab..

[69] Kimura H. (2015). Signaling Molecules: Hydrogen sulfide and polysulfide. Antioxid. Redox Signal..

[70] Nagahara N. (2013). Regulation of mercaptopyruvate sulfurtransferase activity via intrasubunit and intersubunit redox-sensing switches. Antioxid. Redox Signal..

[71] Nagahara N., Nagano M., Ito T., Suzuki H. (2015). Redox regulation of mammalian 3-mercaptopyruvate sulfurtransferase. Methods Enzymol..

[72] Módis K., Asimakopoulou A., Coletta C., Papapetropoulos A., Szabo C. (2013). Oxidative stress suppresses the cellular bioenergetic effect of the 3-mercaptopyruvate sulfurtransferase/hydrogen sulfide pathway. Biochem. Biophys. Res. Commun..

[73] Rao S.P., Xie W., Christopher Kwon Y.I., Juckel N., Xie J., Dronamraju V.R., Vince R., Lee M.K., More S.S. (2022). Sulfanegen stimulates 3-mercaptopyruvate sulfurtransferase activity and ameliorates Alzheimer’s disease pathology and oxidative stress in vivo. Redox Biol..

[74] Parmar M., Grealish S., Henchcliffe C. (2020). The future of stem cell therapies for Parkinson disease. Nat. Rev. Neurosci..

[75] Jurkowska H., Placha W., Nagahara N., Wróbel M. (2011). The expression and activity of cystathionine-γ-lyase and 3-mercaptopyruvate sulfurtransferase in human neoplastic cell lines. Amino Acids..

[76] Kimura Y., Koike S., Shibuya N., Lefer D., Ogasawara Y., Kimura H. (2017). 3-Mercaptopyruvate sulfurtransferase produces potential redox regulators cysteine- and glutathione-persulfide (Cys-SSH and GSSH) together with signaling molecules H_2_S_2_, H_2_S_3_ and H_2_S. Sci. Rep..

[77] Nagahara N. (2020). Activation of 3-mercaptopyruvate sulfurtransferase by glutaredoxin reducing system. Biomolecules.

[78] Wang D., Yang H., Zhang Y., Hu R., Hu D., Wang Q., Liu Y., Liu M., Meng Z., Zhou W. (2021). Inhibition of cystathionine β-synthase promotes apoptosis and reduces cell proliferation in Chronic Myeloid Leukemia. Signal Transduct. Target. Ther..

[79] Bogni A., Cheng C., Liu W., Yang W., Pfeffer J., Mukatira S., French D., Downing J.R., Pui C.H., Relling M.V. (2006). Genome-wide approach to identify risk factors for therapy-related myeloid leukemia. Leukemia.

[80] Chen C.C., Li B., Millman S.E., Chen C., Li X., Morris J.P., Mayle A., Ho Y.J., Loizou E., Liu H. (2020). Vitamin B6 addiction in Acute Myeloid Leukemia. Cancer Cell.

[81] Liu H., Sun J., Guo S., Cheng X., Zhang Z., Wan J., Wang C., Zhi X., Yuan L., Wang H. (2023). Hydrogen sulfide inhibits human T-cell leukemia virus type-1 (HTLV-1) protein expression via regulation of ATG4B. J. Med. Virol..

[82] Shen H., McHale C.M., Haider S.I., Jung C., Zhang S., Smith M.T., Zhang L. (2016). Identification of genes that modulate susceptibility to formaldehyde and imatinib by functional genomic screening in human haploid KBM7 cells. Toxicol. Sci..

[83] Rosado J.O., Salvador M., Bonatto D. (2007). Importance of the trans-sulfuration pathway in cancer prevention and promotion. Mol. Cell. Biochem..

[84] Wang M., Guo Z., Wang S. (2013). The effect of certain conditions in the regulation of cystathionine γ-lyase by exogenous hydrogen sulfide in mammalian cells. Biochem. Genet..

[85] Fernandes D.G.F., Nunes J., Tomé C.S., Zuhra K., Costa J.M.F., Antunes A.M.M., Giuffrè A., Vicente J.B. (2021). Human cystathionine γ-lyase is inhibited by s-Nitrosation: A new crosstalk mechanism between NO and H_2_S. Antioxidants.

[86] Araki S., Takata T., Ono K., Sawa T., Kasamatsu S., Ihara H., Kumagai Y., Akaike T., Watanabe Y., Tsuchiya Y. (2023). Cystathionine γ-lyase self-inactivates by polysulfidation during cystine metabolism. Int. J. Mol. Sci..

[87] Lazarus H., Barell E.F., Oppenheim B.S., Krishan A. (1974). Divergent properties of two human lymphocytic cell lines isolated from a single specimen of peripheral blood. In Vitro.

[88] Iglehart J.K., York R.M., Modest A.P., Lazarus H., Livingston D.M. (1977). Cystine requirement of continuous human lymphoid cell lines of normal and leukemic origin. J. Biol. Chem..

[89] Glode L.M., Epstein A., Smith C.G. (1981). Reduced gamma-cystathionase protein content in human malignant leukemia cell lines as measured by immunoassay with monoclonal antibody. Cancer Res..

[90] Glode L.M., Kriegler M.P., Livingston D.M. (1981). Cysteine auxotrophy of human leukemic lymphoblasts is associated with decreased amounts of intracellular cystathionase protein. Biochemistry.

[91] Kriegler M.P., Pawlowski A.M., Livingston D.M. (1981). Cysteine auxotrophy of human leukemic lymphoblasts is associated with decreased amounts of intracellular cystathionase messenger ribonucleic acid. Biochemistry.

[92] Du S.X., Jia Y.R., Tang H., Sun Y.L., Wu W.S., Sun L.M., Du J.B., Geng B., Tang C.S., Jin H.F. (2014). Immune regulation of hydrogen sulfide in children with Acute Lymphoblastic Leukemia. Chin. Med. J..

[93] Livingston D.M., Ferguson C., Gollogly R., Lazarus H. (1976). Accumulation of cystine auxotrophic thymocytes accompanying type C viral leukemogenesis in the mouse. Cell.

[94] Link D., Drebing C., Glode L.M. (1983). Cystathionase: A potential cytoplasmic marker of hematopoietic differentiation. Blut.

[95] Zhu H., Blake S., Chan K.T., Pearson R.B., Kang J. (2018). Cystathionine β -synthase in physiology and cancer. Biomed Res. Int..

[96] Hellmich M.R., Szabo C. (2015). Hydrogen sulfide and cancer. Handb. Exp. Pharmacol..

[97] Majtan T., Singh L.R., Wang L., Kruger W.D., Kraus J.P. (2008). Active cystathionine β-synthase can be expressed in heme-free systems in the presence of metal-substituted porphyrins or a chemical chaperone. J. Biol. Chem..

[98] Fu M., Zhang W., Wu L., Yang G., Li H., Wang R. (2012). Hydrogen sulfide (H_2_S) metabolism in mitochondria and its regulatory role in energy production. Proc. Natl. Acad. Sci. USA.

[99] Niu W.N., Yadav P.K., Adamec J., Banerjee R. (2015). S-glutathionylation enhances human cystathionine β-synthase activity under oxidative stress conditions. Antioxid. Redox Signal..

[100] Taub J.W., Huang X., Matherly L.H., Stout M.L., Buck S.A., Massey G.V., Becton D.L., Chang M.N., Weinstein H.J., Ravindranath Y. (1999). Expression of chromosome 21-localized genes in Acute Myeloid Leukemia: Differences between Down Syndrome and non-Down Syndrome blast cells and relationship to in vitro sensitivity to cytosine arabinoside and daunorubicin. Blood.

[101] Ge Y., Jensen T.L., Matherly L.H., Taub J.W. (2002). Synergistic regulation of human cystathionine-β-synthase-1b promoter by transcription factors NF-YA isoforms and Sp1. Biochim. Biophys. Acta-Gene Struct. Expr..

[102] Hauck J.S., Moon D., Jiang X., Wang M.E., Zhao Y., Xu L., Quang H., Butler W., Chen M., Macias E. (2024). Heat shock factor 1 directly regulates the transsulfuration pathway to promote prostate cancer proliferation and survival. Commun. Biol..

[103] Ascenção K., Szabo C. (2022). Emerging roles of cystathionine β-synthase in various forms of cancer. Redox Biol..

[104] Zhang W., Braun A., Bauman Z., Olteanu H., Madzelan P., Banerjee R. (2005). Expression profiling of homocysteine junction enzymes in the NCI60 panel of human cancer cell lines. Cancer Res..

[105] Taub J.W., Ge Y. (2005). Down Syndrome, drug metabolism and chromosome 21. Pediatr. Blood Cancer.

[106] Taub J.W., Matherly L.H., Stout M.L., Buck S.A., Gurney J.G., Ravindranath Y. (1996). Enhanced metabolism of 1-beta-D-arabinofuranosylcytosine in Down Syndrome cells: A contributing factor to the superior event free survival of Down Syndrome children with Acute Myeloid Leukemia. Blood.

[107] Ge Y., Jensen T.L., Matherly L.H., Taub J.W. (2003). Transcriptional regulation of the cystathionine-β-synthase gene in Down Syndrome and non-Down Syndrome megakaryocytic leukemia cell lines. Blood.

[108] Zuhra K., Augsburger F., Majtan T., Szabo C. (2020). Cystathionine-β-synthase: Molecular regulation and pharmacological inhibition. Biomolecules.

[109] Kamoun P., Belardinelli M.-C., Chabli A., Lallouchi K., Chadefaux-Vekemans B. (2003). Endogenous hydrogen sulfide overproduction in Down Syndrome. Am. J. Med. Genet..

[110] Panagaki T., Randi E.B., Augsburger F., Szabo C. (2019). Overproduction of H_2_S, generated by CBS, inhibits mitochondrial complex IV and suppresses oxidative phosphorylation in Down Syndrome. Proc. Natl. Acad. Sci. USA.

[111] Canevarolo R.R., de Melo C.P.S., Cury N.M., Artico L.L., Corrêa J.R., Tonhasca Lau Y., Mariano S.S., Sudalagunta P.R., Brandalise S.R., de Zeri A.C.M. (2022). Glutathione levels are associated with methotrexate resistance in Acute Lymphoblastic Leukemia cell lines. Front. Oncol..

[112] Wang J., Liu X., Qiu Y., Shi Y., Cai J., Wang B., Wei X., Ke Q., Sui X., Wang Y. (2018). Cell adhesion-mediated mitochondria transfer contributes to mesenchymal stem cell-induced chemoresistance on T cell Acute Lymphoblastic Leukemia cells. J. Hematol. Oncol..

[113] Fotoohi A.K., Assaraf Y.G., Moshfegh A., Hashemi J., Jansen G., Peters G.J., Larsson C., Albertioni F. (2009). Gene expression profiling of leukemia T-cells resistant to methotrexate and 7-hydroxymethotrexate reveals alterations that preserve intracellular levels of folate and nucleotide biosynthesis. Biochem. Pharmacol..

[114] Lin W., Wang C., Liu G., Bi C., Wang X., Zhou Q., Jin H. (2020). SLC7A11/xCT in cancer: Biological functions and therapeutic implications. Am. J. Cancer Res..

[115] Jyotsana N., Ta K.T., DelGiorno K.E. (2022). The role of cystine/glutamate antiporter SLC7A11/xCT in the pathophysiology of cancer. Front. Oncol..

[116] Lim J.K.M., Delaidelli A., Minaker S.W., Zhang H.F., Colovic M., Yang H., Negri G.L., von Karstedt S., Lockwood W.W., Schaffer P. (2019). Cystine/glutamate antiporter xCT (SLC7A11) facilitates oncogenic RAS transformation by preserving intracellular redox balance. Proc. Natl. Acad. Sci. USA.

[117] Liu N., Lin X., Huang C. (2020). Activation of the reverse transsulfuration pathway through NRF2/CBS confers erastin-induced ferroptosis resistance. Br. J. Cancer.

[118] Lo M., Wang Y.Z., Gout P.W. (2008). The x(c)-cystine/glutamate antiporter: A potential target for therapy of cancer and other diseases. J. Cell. Physiol..

[119] Ghasemitarei M., Yusupov M., Razzokov J., Shokri B., Bogaerts A. (2019). Transport of cystine across xC- antiporter. Arch. Biochem. Biophys..

[120] Koppula P., Zhang Y., Zhuang L., Gan B. (2018). Amino acid transporter SLC7A11/xCT at the crossroads of regulating redox homeostasis and nutrient dependency of cancer. Cancer Commun..

[121] Weisberger A.S., Suhrland G.L. (1956). Studies on analogues of L-cysteine and L-cystine: III. The effect of selenium cystine on leukemia. Blood.

[122] White J. (1941). The effect of dietary cystine on the reaction of dilute brown mice to methylcholanthrene. JNCI J. Natl. Cancer Inst..

[123] White J. (1943). Note on the comparison of dosage of methylcholanthrene on the production of leukemia and sclerotic lesions in strain dilute brown mice on a restricted cystine diet. JNCI J. Natl. Cancer Inst..

[124] White J., Mider G.B., Heston W.E. (1944). Effect of amino acids on the induction of leukemia in mice. JNCI J. Natl. Cancer Inst..

[125] White J., White F.R., Mider G.B. (1947). Effect of diets deficient in certain amino acids on the induction of leukemia in dba mice. JNCI J. Natl. Cancer Inst..

[126] Luo M., Miao Y.R., Ke Y.J., Guo A.Y., Zhang Q. (2023). A comprehensive landscape of transcription profiles and data resources for human leukemia. Blood Adv..

